# Assessing Dungeness River Functionality and Effectiveness of Best Management Practices (BMPs) Using an Ecological Functional Approach

**Published:** 2019-10-01

**Authors:** Eric S. Hall, Robert K. Hall, Sherman Swanson, Wilson Yee, Don Kozlowski, Michael J. Philbin, Daniel T. Heggem, John Lin, Joan L. Aron, Robin J. Schafer, David Guiliano, Eric Wilson

**Affiliations:** 1 USEPA Office of Research and Development, NERL, Systems Exposure Division (SED), Ecological and Human Community Analysis Branch, Research Triangle Park, NC; 2 USEPA Region IX, WTR2, 75 Hawthorne St., San Francisco, CA; 3 Ecology, Evolution and Conservation Biology, University of Nevada, 1664 N. Virginia St., Reno, NV; 4 US Forest Service, Sequoia National Forest, Western Divide Ranger District, Springville, CA; 5 U.S. Dept. of the Interior Bureau of Land Management, Montana/Dakotas State Office, 5001 Southgate Drive, Billings, MT; 6 USEPA Office of Research and Development, NERL, Systems Exposure Division (SED), Ecosystem Integrity Branch, Las Vegas, NV; 7 Aron Environmental Consulting, 5457 Marsh Hawk Way, Columbia, MD; 8 University of Puerto Rico, Río Piedras Campus, 14 Ave. Universidad, Ste. 1401, San Juan, PR; 9 Gulf Coast STORET, LLC, 11110 Roundtable Dr., Tomball, TX

**Keywords:** Best Management Practices (BMPs), Potential Natural Condition (PNC), Proper Functioning Condition (PFC), Total Maximum Daily Load (TMDL), Watershed Condition Framework (WCF), Non-Point Source (NPS), Point Source (PS), Fecal coliform, Ecological function

## Abstract

Effective stream and wetland Best Management Practices (BMPs) restore the physical processes associated with ecological functions to their Proper Functioning Condition (PFC, i.e., the highest attainable ecological status of a riparian area without consideration of economic, administrative, or social constraints). Ecological functions connect stream monitoring and management to mitigate the causes of ecosystem degradation and enhance restoration. The ecological function approach supports sustainable management of many ecosystem services including water quality, water stability (aquifer recharge), and fish and wildlife habitats. The 1993 Forest Ecosystem Management Assessment Team (FEMAT) report listed the Dungeness River as a Tier 1 key watershed, noted that watersheds are the logical spatial unit for ecosystem management, and that watersheds are important in species management, and understanding the interdependence of physical processes. Watersheds are at the spatial scale where physical and biological disturbances can be observed, and where management constraints and planning options for restoration objectives and strategies can be readily assessed. The US Forest Service (USFS) developed a management strategy for the Middle Dungeness River, and in the 1990s, the Upper Dungeness River was listed as impaired due to sediment, which initiated a US Forest Service change to land management practices. The Lower Dungeness River and bay are listed as impaired due to fecal coliform contamination. Assessing and monitoring the drivers of ecosystem function (vegetation, hydrology, soil, and landform) as part of a watershed adaptive management plan, and implementing BMPs to increase ecological functions, will improve aquatic habitat and water quality. Most BMPs, such as Total Maximum Daily Loads (TMDLs), attempt to improve water quality by reducing the amount of external pollutants reaching the impacted waterbodies, but do not focus on improving the watershed functions. The Proper Functioning Condition (PFC) approach is used to examine the condition of wetlands and streams and provide guidance for quantitative approaches (e.g., TMDL, remote sensing) used in watershed restoration. Improving watershed functions is a BMP that facilitates increased flows of water, nutrients, sediment, and other materials, and improves habitat quality. Using improved watershed functions as a BMP, facilitated by the use of remote sensing, TMDLs, and the PFC methodology is a more effective means of reducing risks across a watershed than by using TMDLs alone.

## 1. Introduction

The Clean Water Act (CWA), also known as the Federal Water Pollution Control Act (FWPCA), is a key tool in regulating water pollution [9]. Success of the water programs (e.g., CWA Section 303(d), Total Maximum Daily Load (TMDL) source assessment, and; CWA, Section 319) can be measured by the NPS project funding tracking system called the Grants Reporting and Tracking System (GRTS: Webpage - https://iaspub.epa.gov/apex/grts/f?p=grts:95; Accessed: 12 Oct 2019). GRTS is the central oversight, management, and data-sharing tool for the Section 319 Program, used to assess and prioritize the control of point source (PS) and nonpoint source (NPS) water pollution, and is based on identifying actions for attaining water quality standards through the CWA [9], [10]. The USEPA 2004 National Water Quality Inventory Report to Congress [11] indicates that approximately 44% of rivers and streams, and 64% of lakes and reservoirs surveyed are impaired for fishing, swimming, and other uses. The primary causes of impairment are pathogens, habitat alterations, nutrients, and other factors. This report raises some interesting questions about the USEPA’s water quality program, and its ability to deal with NPS pollution. After more than 35 years of implementing the CWA and investment of billions in public [12] and private funds [13], states acknowledge that NPS pollution is the most important remaining cause of water quality problems (Webpage: https://www.epa.gov/nps/basic-information-about-nonpoint-source-nps-pollution; Accessed 12 Oct 2019).

### 1.1. Ecosystem Management Considerations

Bernhard et al., [14] noted that funding [12], [13] for improving quality and in-stream habitat does not provide the necessary data and information for evaluating the ecological effectiveness of restoration activities. The questions for state and tribal environmental managers are: how to assess and prioritize water quality in areas (e.g., the Upper Reese River in Nevada [7], or in Illinois, Pennsylvania, Maryland, Virginia, and West Virginia [15] where implementation of Best Management Practices [BMPs] is needed); what are the most effective BMPs [16], and; how to monitor BMP effectiveness [6], [8]. Giri et al., [17] evaluated targeted methods for BMP implementation, and found BMP efficiencies vary, depending on design, maintenance, and placement within the watershed [4], [15], [18], and [19]. Physically, ecosystems are dynamically affected by biogeoclimatic setting and anthropogenic modification of natural conditions. Changes in ecological condition, affect the chemical and biological community structure, causing further alterations to the physical, chemical, and biotic environment. Variability, in ecosystem structure and function and in pollution sources, impacts the effectiveness of potential environmental solutions [17].

Sources of pollution can be external (point sources and some nonpoint sources, such as runoff of anthropogenic chemicals) or internal, expressed as altered concentrations of naturally occurring materials (e.g., decreased flow, channel incision, etc.). These pollution sources result in an ecosystem with altered functioning of its physical processes. Effective solutions focus on the drivers of ecosystem functioning (vegetation, hydrology, soil, and landform). External pollutants/stressors create problems with water quality (e.g., nutrients, trace metals, temperature, pH, dissolved oxygen) and/or habitats (substrate, water depth, velocity, temperature, insolation, etc.) [16]. An appropriate management strategy incorporates ecosystem patterns, attributes, and processes to sustain ecosystem functions, and support biological diversity, including a range of environmental processes and associated variations.

The central focus of an ecosystem management strategy should be on physical processes, emphasize ‘self-healing’, and prioritize actions which maintain, and restore, the necessary conditions for ecosystem resilience and restoration. Most states and tribes prioritize their waterbodies for TMDL development, and/or BMP implementation, using a statewide scheduling process, that reviews and assesses water quality problems listed on the most recent CWA Section 303(d) list of impaired waters for their state or tribal region. The objective of this process is to enhance, and accelerate, efforts to restore impaired water quality through watershed planning, and implementation of TMDLs. It is important to obtain stakeholder involvement in developing TMDLs, and implementing BMPs [10]. However, without the subsequent step of determining the relative contribution of external versus internal (altered concentration of natural materials) pollutants, and the driving mechanisms causing altered concentrations, any planned restoration activities (corrective actions), undertaken as a result of this process, may be incomplete, and could be ineffective. USEPA developed the Nitrogen and Phosphorus pollution Data Access Tool (NPDAT), to assist states and tribes in these efforts (Webpage: http://water.epa.gov/scitech/swguidance/standards/criteria/nutrients/npdat_index.cfm - Accessed: 12 Oct 2019). As seen in the literature, the majority of BMPs focus on water quality and external pollutants [4], not on watershed functions [5]. Thus, most of the state and tribal approaches are based on how the ecosystem functions, its resiliency to perturbation, or its ability to assimilate stressors. However, it is the adjustments in watershed (ecological) functions that provides resilience, through preventing, and, when required, processing surges in water, nutrients, sediment, and other materials, and by improving habitat quality [6], [7], and [8]. The quality of a stream, and wetland riparian ecosystem, directly relates to the adjacent uplands, and the ‘up-stream’, and ‘down-stream’ conditions. Improved knowledge of aquatic and upland interactions, solely focused on the riparian zone at local watershed scales, is essential in evaluating and designing land management approaches for improving stream and riparian wetland resources.

### 1.2. Best Management Practices (PFC, Remote Sensing, TMDL)

Wyman, et al., [20] indicate that water quality, and channel morphology measurements, are not appropriate tools to evaluate BMP strategy changes in the short-term. Significant assimilative capacity of a stream and wetland stems from riparian vegetation, especially obligate vegetation [7], [21], [22], [23], and [24]. Vegetation attribute measurements may be more appropriate, because they are the first to react to stress, and are drivers of ecosystem change. Water quality, and aquatic community structure, depend on the amount, and timing, of water flowing through the system. Hydrology also depends on climate, weather, and sometimes water rights (which are outside of a manager’s control). Consequently, ecosystem improvement, and the adequacy of a management strategy (i.e., BMPs), can be understood by monitoring riparian conditions, and the attributes important in sustaining ecosystem functionality. Understanding the expected recovery rates, for a specific riparian area, helps in developing achievable objectives, that can be met, within a designated timeframe, through adaptive management.

Prichard et al., [22] defines the Properly Functioning Condition (PFC) of a stream, and wetland riparian ecosystem, as having the ability to: “Dissipate stream energy associated with high water flows, thereby reducing erosion and improving water quality; filter sediment, capture bedload, and aid floodplain development; improve floodwater retention and groundwater recharge; develop root masses that stabilize streambanks against cutting action; develop diverse ponding and channel characteristics to provide the habitat and the water depth, duration, and temperature necessary for fish production, waterfowl breeding, and other uses, and; support greater biodiversity.” This is the most desirable condition, and the highest PFC rating, that a stream or wetland riparian system can attain.

The purpose of this paper is to: a) explain the concept of the Proper Functioning Condition (PFC) methodology [24] in assessing stream and wetland attributes, prioritizing resources, and providing a context for quantitative approaches; b) illustrate the importance of Remote Sensing (along with hydrographs and reports) in tracking and documenting change that can be assessed by PFC; c) describe the impact of regulatory, educational, and management action in improving Total Maximum Daily Load (TMDL) levels (fecal coliform), and; d) explain how PFC, Remote Sensing (along with hydrographs and reports), and TMDL can be used together as a Best Management Practice (BMP) in assessing the functionality of riparian-wetland areas.

## 2. Methods

### 2.1. Study Area

The Dungeness River headwaters are in the northeastern portion of the Olympic National Forest, and discharge into Dungeness Bay, and the Strait of Juan de Fuca, on the eastern side of Sequim WA (Figure 1a and Figure 1b). The Lower and Middle Dungeness River are low to moderate gradient sinuous (wandering back and forth across the floodplain, in an ‘S-shaped’ pattern) stream channels. The Dungeness River Restoration Work Group (DRRWG) [39] identified excessive flooding, and declining stocks of salmon, to be the result of loss of important physical processes and riparian functions. The DRRWG Report [39], indicated that protection of self-sustaining salmon stocks in the Dungeness River required an approach that recognized, and restored, important river functions relevant to local potential.

### 2.2. Assessment Strategy/Approach

Assessing stream and wetland functionality involves determining a riparian area’s potential using local knowledge (historic photographs, survey notes, and/or documents indicating historic condition), species lists (historic animals and plants), soils, hydrology, geomorphology, identifying currently existing vegetation, monitoring of pollutants and pathogens, and any limiting factors (human-caused and natural), to determine if they can be corrected. Most of these limiting factors can be rectified through proper management [6], except for permanent construction (e.g., dams, diversions, permanent channel modifications), which is not as easy to modify, since the placement of permanent structures can result in a stream-wetland area’s flow regime being modified to a new (altered) potential. In this study, PFC, TMDL, and remote sensing (along with hydrographs and historical report information) are used together to develop a more complete picture of the status of the area and how to improve the functionality of the riparian-wetland area.

### 2.3. Total Maximum Daily Load (TMDL)

The area between Port Angeles and Sequim, Clallam County WA experienced 16% growth between 1990 and 2000. Fecal coliform bacteria has been increasing in Dungeness Bay since 1997 [37]. In May 2000, the Washington Department of Health closed 300 acres of Dungeness Bay to commercial shellfish harvesting [38]. In May 2001, the Washington Department of Health added another 100 acres to the closure area, because bacteria concentrations exceeded the State and Federal water quality standards.

TMDLs focus on quantitative water quality measurements, and their impacts to human health and aquatic communities [25]. When dealing with NPS pollution (e.g., sediment, nutrients, pathogens), water quality and aquatic organisms are response (lagging) indicators [25], [26], and [27]. To address aquatic impacts from environmental stressors, it is important to understand the drivers of ecosystem function (vegetation, hydrology, soil, and landform), and recognize their role in the capture, storage, and safe release of water, sediment, nutrients, and organic materials. TMDL data was used to assess the impact of state and tribal corrective actions on fecal coliform levels.

### 2.4. Proper Functioning Condition (PFC)

Proper Functioning Condition (PFC) is a qualitative, science-based approach, used to assess stream and wetland hydrologic, vegetative, and geomorphic attributes and processes accomplished by a multidisciplinary/interdisciplin ary team (ID team), at a point in time [24], [28], [29], and [30]. Natural riparian-wetland areas are best understood through the interactions between the vegetation, hydrology, soil, and geology of those areas. Therefore, assessing the functionality of a riparian-wetland area requires an interdisciplinary (ID) team, including specialists in vegetation, hydrology, soils, and geology. The team must include a biologist who understands the fish and wildlife in the specific riparian wetland area(s) being studied. PFC is also an appropriate starting point for determining, prioritizing, and inventorying riparian resources, developing monitoring needs [31], and providing context for quantitative data (e.g., TMDLs). An ID team must understand stream dynamics, and potential, and use their professional experience, and judgment, to accurately complete a qualitative assessment [28]. Use of quantitative data and techniques (i.e., field measurements, [32], [33], and remote sensing [34]) is encouraged for individual, or team calibration, or where opinions may differ [24].

The term PFC is both a methodology, used to assess the (physical) functioning of riparian-wetland areas, and a description of the (most desirable) condition of riparian-wetland areas. The PFC assessment methodology is defined in [28]. The Watershed Condition Framework (WCF) methodology is defined in [35] as a structured, integrated approach, for implementation, when restoring grasslands, national forests, and priority watersheds. The technical guidance for implementing the WCF methodology is defined in [36]. The PFC has 17 ecosystem indicators/attributes, grouped under 3 categories (hydrology, vegetation, and erosion/deposition) for lotic (rapidly-moving fresh water) ecosystems. The WCF has 12 watershed condition indicators, grouped under 4 categories (aquatic-physical, aquatic-biological, terrestrial-physical, terrestrial-biological). Despite the differences, the two methodologies provide overall assessments that align with each other as shown in Table 1. Given the more abundant literature on the PFC methodology, that is the approach which was selected for this analysis.

Riparian Proper Functioning Condition (PFC) refers to how well the physical processes within a stream, and wetland riparian area, are capable of sustaining a state of resiliency [20], [24], [29], and [30]. This resiliency allows an area to provide and produce desired, and valued, ecosystem services (e.g., fish habitat, livestock and/or wildlife forage, water purification, carbon storage and nutrient cycling) over time [20]. A PFC rating for a lotic (rapidly-moving fresh water) ecosystem [24], and a lentic (still, fresh water) ecosystem [30], relates how well the physical stream/riparian processes are functioning in relation to its Potential Natural Condition (PNC). PFC is determined by assessing the physical processes of riparian-wetland areas through consideration of hydrology, vegetation, and soil/landform attributes appropriate for the potential of the lentic (or lotic) ecosystem. By focusing on physical functioning, the PFC protocol is designed to yield information about the biology of the plants and animals dependent on the riparian-wetland area [29], [30]. PFC provides information indicating how well a riparian-wetland area is physically functioning, and facilitating the maintenance or recovery, of desired attributes [24]. The last column of Table 1 contains the indicators (i.e., vegetation, geology, soil, water) used by the Interdisciplinary (ID) Team specialists to assess and provide ratings of watershed health/status. The best way to explain the full meaning of PFC comes from [28]: “Riparian-wetland areas are functioning properly when adequate vegetation, landform, or large woody debris is present to:

1) dissipate stream energy associated with high waterflows, thereby reducing erosion and improving water quality;
2) filter sediment, capture bedload, and aid floodplain development;
3) improve flood-water retention and ground-water recharge;
4) develop root masses that stabilize streambanks against cutting action;
5) develop diverse ponding and channel characteristics to provide the habitat and the water depth, duration, and temperature necessary for fish production, waterfowl breeding, and other uses;
6) and support greater biodiversity.”

The PFC rating, ‘Functional-At-Risk’ (FAR), refers to riparian areas that are functioning, but have an existing soil, water, or vegetation attribute which makes them susceptible to future degradation. There are three sub-types of FAR designations, ‘Trend Up’, Trend Down’, and ‘Trend Not Apparent’. A FAR sub-rating is an assessment of direction of change (e.g., upward or downward) in condition either towards, or away from, its potential or functionality (PNC) [29], [30]. The trend is determined by comparing the present condition with previous photos, trend studies, inventories, other documentation, or personal knowledge. The lack of historical information on the condition of a site may lead to a “trend not apparent” assessment for the FAR PFC rating, unless other clues are well understood such as the population growth of young woody species (e.g., willows). The PFC rating, ‘Non-Functioning’ (NF), indicates the stream and wetland riparian area is in a degraded state. The Middle Dungeness River has a Proper Functioning Condition (PFC) rating of ‘Functional-At-Risk’ [2], however, that rating does not denote a trend, nor a determination, on whether the river is improving or degrading. The PFC methodology was used to provide a qualitative assessment of the functioning of the riparian area and the prevailing trend(s), where applicable.

### 2.5. Remote Sensing and Supporting Data Sources

The analysis of the Dungeness River included remote sensing imagery from the US Geological Survey (USGS) and US Department of Agriculture [40]. Because of the resolution of the imagery, only the mainstem of the Dungeness River, and the larger tributaries (Matriotti Creek and Meadowbrook Creek) in the Lower Dungeness River, were analyzed using the PFC protocol [22], [24], [34], [40], and [41]. Supporting data sources also included USGS hydrologic and water quality data, for the Dungeness River, and the US Environmental Protection Agency (USEPA) Storage and Retrieval (STORET) data management system data, for the entire state of Washington. The Dungeness River was divided into three reaches (i.e., level, uninterrupted lengths with similar hydrological conditions), Upper (upstream of the USFS boundary), Middle (USFS to US 101), and Lower (US 101 to shoreline) (Figure 1a), and matches the USFS [42] assessment of the Upper Dungeness River, and Middle Dungeness River [2]. The remote sensing and historical reports were used to display and describe the changes over time caused by human activity and provide context for the overall assessment.

## 3. Results

### TMDL Assessment (Fecal Coliform) –

To address the elevated bacteria concentrations, the state of Washington developed a Total Maximum Daily Load (TMDL) for fecal coliform in the Lower Dungeness River and Matriotti Creek [38]. Subsequent water quality evaluations show mixed results. Significant impacts on the river come from animal and human wastes, resulting from hobby farming, and poorly maintained septic systems [37]. Woodruff et al., [47] noted that by late 2009, the Washington Department of Health-initiated closures of the Dungeness Bay to shellfish harvesting were still in effect, indicating that fecal coliform contamination was still a problem. In the Dungeness River watershed, local and regional institutions have worked collaboratively for over 20 years to maintain and restore ecosystem functions [47]. With continued urban expansion, a variety of watershed ecosystem problems have continued and expanded (e.g., storm water runoff, excess sediment, impaired in-stream flows). Issues include the listing of salmonid species under the Endangered Species Act, and the closure of the Dungeness Bay to shellfish harvesting beginning in 2000 [37], [38], due to high levels of fecal coliform (FC) bacteria. These events indicate continued degradation of the Dungeness River ecosystem functions.

**Figure 2.**
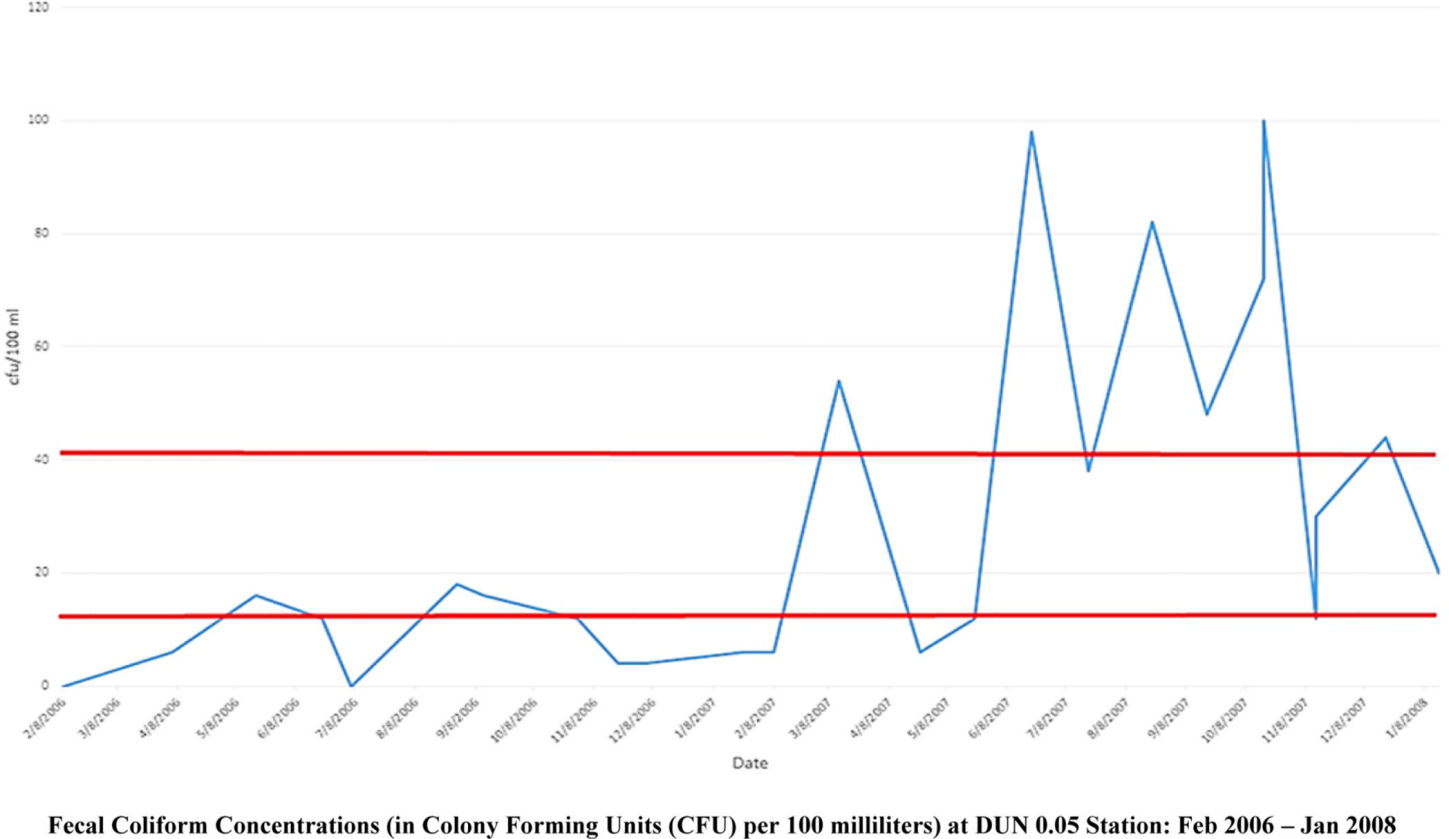
Fecal coliform concentrations at the State of Washington monitoring station, DUN 0.05, at the mouth of the Dungeness River (Monthly sampling from February 2006 to January 2008). To protect shellfish harvesting in the Dungeness Bay, the Dungeness River fecal coliform standard is 13 colony forming units (cfu)/100 ml (lower red line), and should not exceed 43 cfu/100 ml (upper red line), where cfu represents the colony-forming units of bacteria found in a sample. The tributaries to the Dungeness Bay should meet the current Class AA freshwater standard of a geometric mean of 50 cfu/100 mL [38]

In 2004 the Dungeness Bay was also listed as impaired by fecal coliform. The TMDL implementation plan was documented in the Water Cleanup Plan for Bacteria in the Lower Dungeness Watershed [37]. The predominant sources of fecal coliform contamination in freshwater and marine environments were, in rank order, avian (19.6%), gull (12.5%), waterfowl (9.7%), raccoon (9.2%), unknown (7.3%), human-derived (7.1%), rodent (6.3%), and dog (4.3%) [47]. Bird groups combined represented ~42% of the samples collected and analyzed [47]. Wildlife represented ~26% of isolates, and domestic animal and livestock groups each represented approximately 7% of isolates [47]. Matriotti Creek (MAT0.1) had the highest frequency of occurrence of human sources, occurring in ~70% of samples collected and analyzed [47]. A subsequent *Bacteroides fragilis* (bacteria generally found in the human colon) Polymerase Chain Reaction (PCR) genetic profile study detected human and/or ruminant-derived source bacteria, indicating a wider spread of these sources throughout the lower watershed [47]. The TMDL-associated restoration activities (corrective actions) included piping of irrigation ditches, pasture management, manure storage, pumping and repair of septic systems, and outreach and education efforts with area residents. The Washington Department of Ecology targeted private landowners, because they were able to use their regulatory authority, and apply federal and state resources, to the restoration efforts. The Jamestown S’Klallam Tribe, and Clallam County, monitored fecal coliform bacteria levels at selected TMDL monitoring sites to determine the effectiveness of the restoration activities/corrective actions (Figure 2). The TMDL restoration activities continue to the present day.

**Figure 3.**
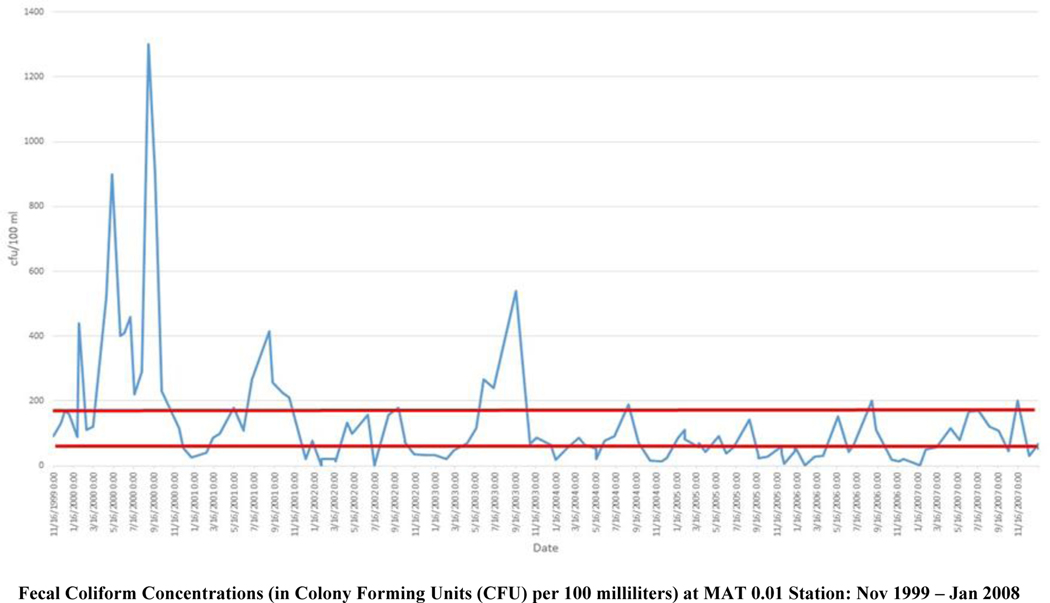
Fecal coliform concentrations at the State of Washington monitoring station, MAT0.01, at the mouth of the Matriotti Creek (Monthly sampling from November 1999 to January 2008). To meet the TMDL target in the Dungeness River, the fecal coliform bacteria concentrations for the Matriotti Creek needed to meet a water quality standard of 60 cfu/100 ml (lower red line), and not exceed 170 colony forming units (cfu)/100 ml (upper red line), where cfu represents the colony-forming units of bacteria found in a sample

The Washington Department of Ecology analyzed the post-TMDL corrective action data that the Jamestown S’Klallam Tribe collected, and found significant improvements in bacteria levels in some areas. The Matriotti Creek fecal coliform levels significantly improved. Several sites met the bacteria target levels set for the creek by 2004, and all sites monitored showed some improvement (Figure 3). The mouth of the Matriotti Creek needed only a 38% further reduction in fecal coliform levels, which is a significant improvement over the 78% reduction that was needed in the year 2000 [38].

However, the Dungeness River has not yet met the TMDL targets. Some improvements in fecal coliform levels were seen during the irrigation season, but these could have been due to decreasing bacteria levels in Matriotti Creek. The Meadowbrook Creek fecal coliform concentrations increased slightly between the completion of the TMDL corrective actions in 2002, and the evaluation study in 2004 [38]. As reported in 2010 [48], the TMDL efforts did reduce fecal coliform loads, but 70% of the watershed still has not met water quality standards. Also, salmon stocks have not improved significantly in the watershed [2]. Therefore, targeting agricultural lands did have an impact in decreasing the release of fecal coliform, but not enough to continuously meet water quality standards. The current federal regulation governing fecal coliform standards [49] is available.

**Figure 4.**
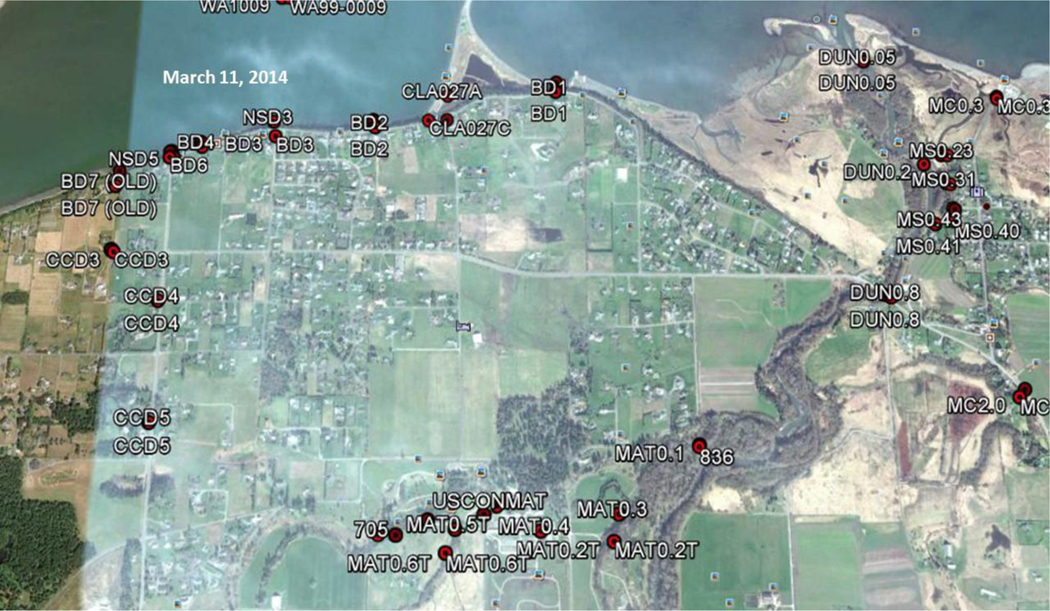
USDA National Agriculture Imagery Program (NAIP) March 11 2014, image of the confluence of the Matriotti Creek with the Dungeness River. Red dots are the State of Washington sampling locations. The MAT0.1 sampling site is located in the lower center-right of the image (Note the narrow riparian area along the Matriotti Creek.)

As seen in Figure 2, the fecal coliform concentrations at the mouth of the Dungeness River were low, then subsequently in early 2007, fecal coliform concentrations begin to increase. In Matriotti Creek (Figure 3), the TMDL-associated corrective actions show an impact in lowering the fecal coliform concentrations (except for mid-to-late-summer spikes). However, on average, Matriotti Creek has exceeded its water quality standard of 60 cfu/100 ml. The key to managing fecal coliform is to prevent the bacteria from reaching the stream. In the Dungeness River floodplain, connectivity is essential for long term sustainability (Figure 4). As seen in Figure 4, the Matriotti Creek, which is in the bottom center of the image, has a narrow riparian area, with agricultural fields and residences adjacent to the stream channel. The Dungeness River has shown improvement in the riparian area over time, however, Matriotti Creek has not. Therefore, establishment of ‘natural’ stabilizing vegetation is essential to maintain the appropriate hydraulic geometry, and long-term stream bank stability.

Restoration strategy for pollution control and maintaining healthy aquatic habitats often depends on managing land to facilitate the natural recovery of riparian functions. Osborn and Ralph [51], found the physical processes of the Dungeness River floodplain had been altered by human activities (i.e., diking, bridge and road constrictions, removal of log jams and large woody debris, forest and agricultural land management, and water withdrawals). These in-stream activities resulted in the steep decline in the Dungeness River salmon populations. The Puget Sound Cooperative River Basin Team [52], reported that the Dungeness River watershed salmon populations did not follow similar trends in the Puget Sound Basin. The decline is a result of events occurring specifically in the Dungeness River rather than from regional factors [52].

The focus was on the functional changes occurring within the Dungeness River watershed, Sequim WA, before and after the USFS’s change in land management strategy from ‘clear-cutting’ to ‘forest thinning’ [42], which was ultimately replaced by the 1994 NWFP [1]. This led to a substantial reduction in timber harvesting, additional stream and riparian protections, and an emphasis on watershed restoration, especially the decommissioning of unstable, ‘high risk’ roads, and has been an excellent first step in reducing sediment load into the Dungeness River. As seen in Figures 7–10, sediment and nutrients can come from the materials stored in riparian areas and wetlands. Loss of ecological function and physical form unraveled the assimilation processes, resulting in the loss of fish habitat. Wetlands are used for water quality remediation, because of their ability to sequester pollutants. Riparian wetlands provide a barrier for overland flow, and dissipate energy, allowing sediment deposition, and create aquatic and riparian habitat complexity. The degraded condition of the Matriotti Creek riparian area, as seen in Figure 4, prevents the natural assimilative capacity of the ecosystem from achieving the required water quality standards and providing a high-quality aquatic habitat.

### PFC Assessment –

The ecosystem function potential of the Middle Dungeness River was identified [2] as having moderate channel sinuosity, stabilizing herbaceous and woody obligate vegetation, and containing large woody debris. This assessment [2] is similar to an 1858 surveyor’s field notes, which indicated that a forested ecosystem existed along the entire length of the Dungeness River and wetland areas [43]. Therefore, the Desired Natural Condition (DNC) of the Dungeness River watershed is to be a resilient, and properly functioning watershed, with a complex aquatic riparian and terrestrial forested ecosystem [2]. The functionality of the three reaches of the Dungeness River is discussed with respect to hydrology, vegetation, soil, and landform attributes/characteristics are assessed using the PFC methodology.

The PFC approach provides the ‘big picture’ assessment of a riparian-wetland area, is descriptive, and can suggest which adaptive management strategies can be applied to improve an area. Using the PFC methodology, all three reaches of the Dungeness River were assessed as being in different stages of ‘Functional-At-Risk’ (FAR) status [24]. Hydrology and vegetation show a slight decrease in functionality in the downstream direction. The primary issue for all three reaches is related to erosion and sediment deposition. A high sediment supply may be a result of the easily eroded country rock of uplifted, poorly consolidated, marine sediments, and basaltic crustal material, where human activity is not a factor. The three reaches are still ‘functional’, but could become non-functional if prudent actions are not taken.

The Upper Dungeness River Reach is located on USFS land. This reach was assessed as being in the higher end of FAR status (shown as green in Figure 1a). The Upper Dungeness River Reach is not in balance with the water and sediment being supplied, and the revegetation of its point bars is slow.

The Middle Dungeness River Reach extends from the USFS boundary to US Highway 101, and was assessed as being in the middle range of FAR status (shown as yellow in Figure 1a). The channel is evolving, and is expanding its flood plain within the incised channel [24], [44]. Excess sediment and water are inhibiting the ability of the vegetation to stabilize both the point and middle channel bars. Also, excess sediment has caused the stream to braid (develop small temporary islands), and in some cases, to have an anastomosed (connected) channel pattern [45]. However, the riparian zone is widening, and the vegetation is an excellent source of large woody debris, which is useful in trapping sediment, diverting high and low flows, and in providing cover and shade for aquatic organisms [54].

The Lower Dungeness River Reach extends from US 101 to the shore zone, and was assessed as being in the middle one-third of FAR status (shown as light yellow in Figure 1a). North of US 101, the stream channel becomes increasingly less braided/anastomosed, and forms a single channel. The point bars are being revegetated.

Matriotti Creek and Meadowbrook Creek were assessed as being in the low end of FAR status to ‘Non-Functioning’ (shown as red in Figure 1a). Both creeks have been channelized because of agricultural practices and urbanization, which has reduced their sinuosity (ability to wander back and forth across the floodplain, in an ‘S-shaped’ pattern), and the streams’ ability to migrate laterally. Riparian areas are narrow, and appear to be inadequate to protect banks, and dissipate energy, during high flows. Human activity has caused this situation, and proper management can be used to improve the trends.

In late summer and early fall, anadromous fish runs in the Dungeness River are dependent on higher seasonal stream flows [46]. An article in the Peninsula Daily News by Jeff Chew, (September 21, 2011, http://www.peninsuladailynews.com/news/dungeness-river-salmon-run-biggest-in-10-years-just-in-time-for-river-festival-video/; Accessed: 12 Oct 2019) indicated that (at that point in time) the Dungeness River salmon run was the biggest in 10 years. As seen in the September base flow (Figure 6), the discharge was high, allowing the salmon to move upstream. In September 8, 2013, Arwyn Rice, Peninsula Daily News (http://www.peninsuladailynews.com/news/chinook-salmon-return-to-elwha-river-to-spawn-upstream-from-location-of-former-dam-updated/; Accessed: 12 Oct 2019) reported (at that point in time) it was the biggest salmon run in a half-century.

The USFS [2] rated the Upper Dungeness River as being ‘Functional-At-Risk’ (FAR), but did not further specify the details of that rating. The results of this analysis are more specific, and indicate that the Upper Dungeness River Reach is continuing to improve to the present day (green box – Figure 1a). The Middle and Lower Dungeness River Reaches are also in a FAR condition (dark yellow box, and light yellow box in Figure 1a, respectively), with an improving (upward) trend. Today, the channel has developed a new flood plain within the channelized/incised section of the river [24], [44]. Riparian vegetation is populating and stabilizing the point bars, and increasing surface water flow later into the year. However, the tributaries (Matriotti Creek, Meadowbrook Creek) in the Lower Dungeness River are at the low end of FAR (red boxes in Figure 1a), or in a ‘Non-Functioning’ condition, with no apparent trend, indicating that these tributaries are either not improving, or are further degrading. It should be noted that any remote sensing, performed in conjunction with PFC analysis, should complement the ‘on-the-ground’ assessment.

### Remote Sensing (using Hydrographs and Historical Reports) to Assess Morphological Changes –

**Figure 5.**
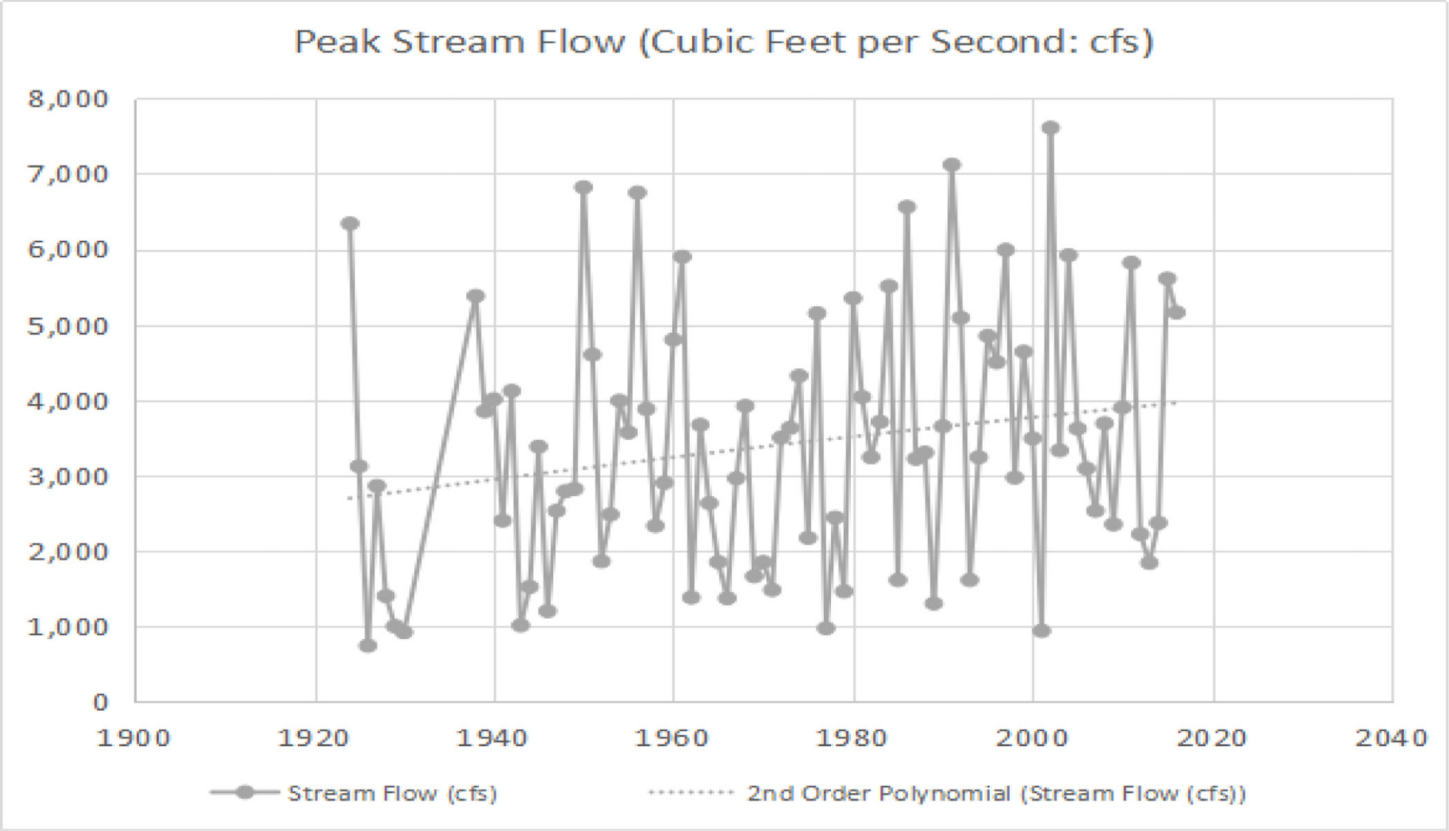
Dungeness River USGS hydrograph of peak flow from 1924 to 2016 (in cubic feet per second [cfs]). The trend in peak flow has increased over time. **Note:** 1000 cfs = 28.32 cubic meters per second

Understanding the long-term evolution of a riparian-wetland area provides the basis for what actions should be taken to restore functionality when required. The first settlers to the Dungeness River watershed noted the area was heavily forested, from its shoreline to the Olympic Mountains. Collins [43] reported the Dungeness River delta area increased in the early 1900’s, indicating a large sediment load moving into the coastal zone. Possible sources of the sediment load include forestry practices, conversion of forested areas to agricultural areas, and/or incision of the Dungeness River channel [55]. Growth of the delta gradually diminished from the 1930’s to 1970’s, and had no net change from the 1970’s to 2000 [43]. A possible explanation for the decreasing delta could be a result of the river channel recovering its riparian ecosystem, and trapping sediment in the channel. An additional explanation could be attributed to channelization and flow diversions of the Dungeness River, and the draining of freshwater wetlands, that began in the early 1960’s [2], [39], and [43]. Channelization projects, such as levee construction, has led to erosion and decreased sediment [56], and wetland drainage can react in a similar manner. Flow diversions are effective in removing water from an area, but in some situations, they block the movement of sediment [57]. The hydrograph of peak flow for the Dungeness River from 1924 to 2016 is shown in Figure 5, with the average peak flow being approximately 2800 cubic feet per second (79.3 cubic meters per second) in 1924 and 4000 cubic feet per second (113.3 cubic meters per second) in 2016.

**Figure 6.**
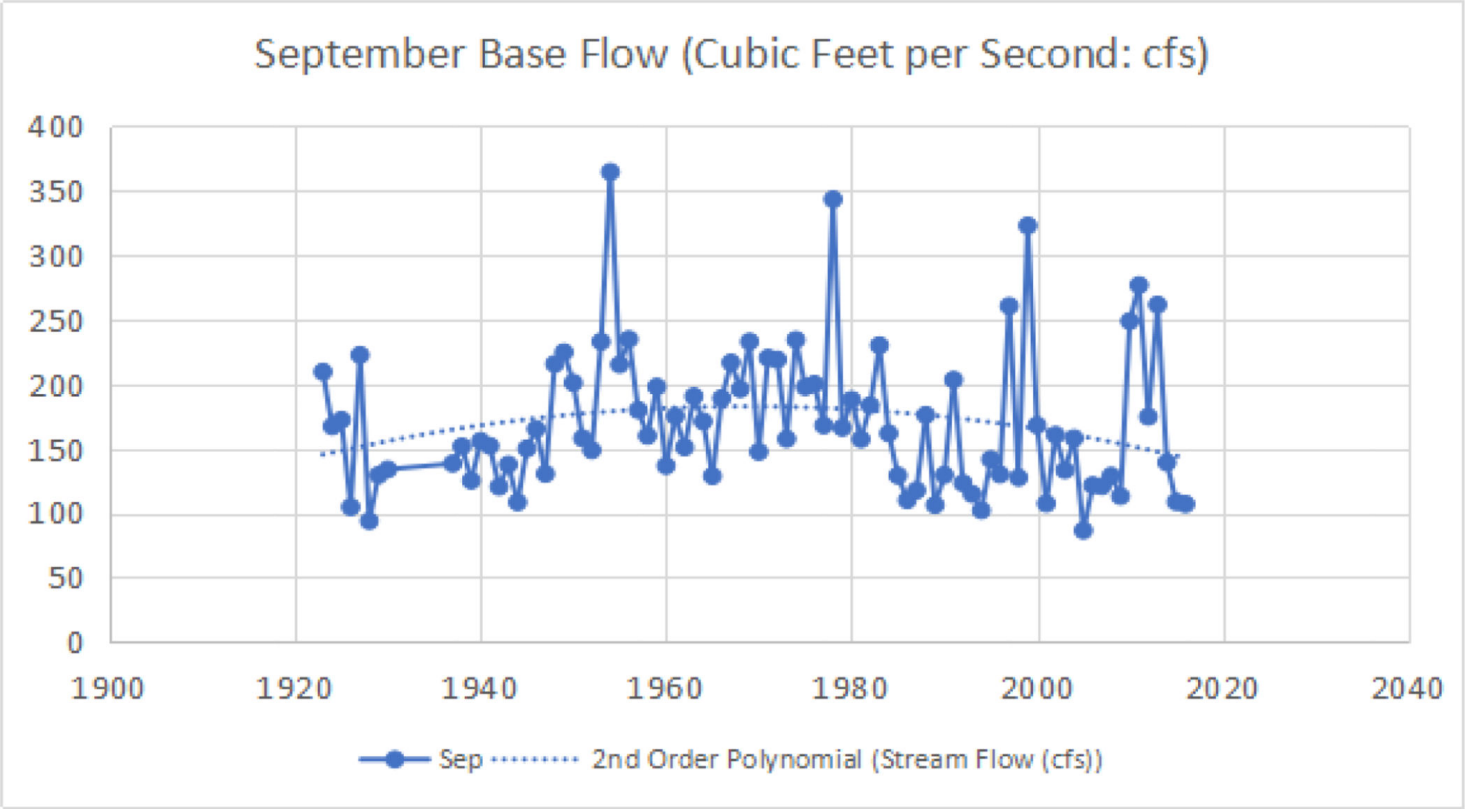
Dungeness River late summer (September) base flow USGS hydrograph from 1924 to 2016 (in cubic feet per second [cfs]). The annual base flow discharge increases from 1924 to 1970. Except for peak discharge years in 1955, 1978, 1997, and 1999, the variance of the base flow discharge increases over time, but is fairly stable. However, the discharge decreased after 1982, when the hatchery fish rack was removed, and this is illustrated by the (dotted) polynomial trend line. After 1982, the September base flow decreased until 2010, possibly due to climatic changes resulting in drought conditions. From 2010 to 2013, the September base flow increased, as shown by the solid line, possibly indicating a change in improved riparian functional condition, with more water being retained in the watershed, and released slowly over time. **Note:** 100 cfs = 2.832 cubic meters per second

The annual base flow discharge for the Dungeness River, between 1924 to 2016 in September, increased between 1924 to 1970, is stable, and indicates peak discharge years in 1955, 1978, 1997, and 1999. After the hatchery fish rack was removed in 1982, the discharge decreased, and subsequently the September base flow decreased until 2010, and increased between 2010 to 2013. A decrease in watershed storage capacity, with a decreased end of summer (and early fall) base flow for 1924 through 2016, is shown in Figure 6, and the effects of the alterations are seen as an increase in annual flow, indicated by the (dotted) polynomial trend line, in Figure 5. The channelization and draining of the wetlands directed more of the flow into the stream. These modifications were designed to continuously move water through the river. The net result was a continuously degrading, but functional ecosystem, and a loss of aquatic and wildlife habitat.

The USFS in their Dungeness River Sub-Watershed Restoration Plan [2] noted that prior to 1994, ‘clear-cut’ logging and roads heavily influenced channel characteristics (shown in Figure 8). As seen in Figure 8A (June 1975) and Figure 8B (May 1987), stream channelization, forestry practices, and other anthropogenic activities, led to stream channel degradation. The buildup of excess sediment (seen as light-colored streaks/areas in the stream channel in Figure 8B) is shown, and is not being moved through the system. The stream channel, as shown in the top red box in Figure 8B, is straighter, north of US Highway 101 (appearing as a faint pink line through the center of the image), and has widened (Figure 8B). Figure 8 shows the impact of land use at two distinct points in time.

The long period of channel alterations along the Dungeness River, and within the watershed, increased peak flow into the channel, and also had an impact on the annual average discharge (Figure 8). The annual average discharge of the Dungeness River increased into the 1980’s. Low water years in the mid-to-late 1980’s slowed the transport of material through the river (Figure 8B), but peak flows were still high. From 1994, the physical dynamics of the watershed changed (Figures 9–12).

As seen in Figure 9, the pattern of annual average discharge mirrors annual rainfall until the year 2000, when annual discharge shows less variability than annual rainfall. This also matches the September stream flow conditions in Figure 6.

As seen in Figure 10, the Lower Dungeness River (Figure 10A) is channelized, and has multiple outlets to the bay. The delta and barrier spit appear to be expanding from excess sediment. The Middle Dungeness River (Figure 10B), north and south of US Highway 101, is an incised, braided stream channel, with little to no re-vegetation of the point bars. The braided stream channel indicates an excessive amount of sediment is being supplied from upstream. As seen in Figure 10, the sediment in 1994 (Figure 10B) dominates the channel morphology (as compared to Figure 11A and Figure 11B). The practice of ‘clear cutting’, and the friable mountain soils, are most likely causing mass wasting events, and/or consistent and chronically increasing sediment rate. The sediment also contributes to the expansion of the barrier spit at the mouth of the river (Figure 10A).

**Figure 7.**
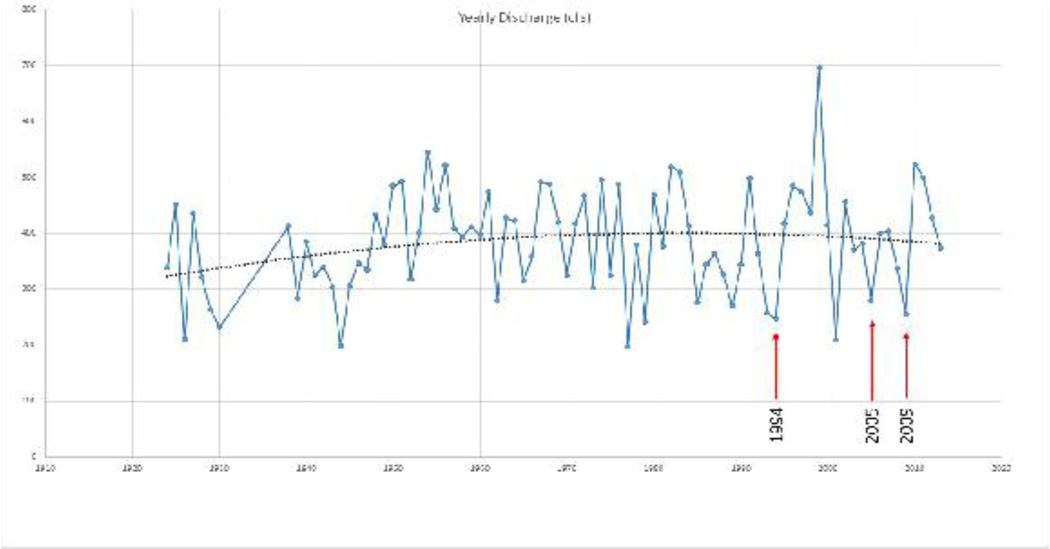
The hydrograph of annual discharge of the Dungeness River from 1924 to 2013. The annual average discharge (solid line) increases from 1924 to approximately 1985. The hydrograph average (dotted polynomial trend line) shows a slight decrease from 1985 to 1995, continuing to decrease until the present. Lower variability in annual discharge in the hydrograph after 2005 indicates an improved riparian functional condition, with more water being retained in the watershed. The red arrows indicate dates of satellite imagery corresponding to what is shown in Figures 10, 11, and 12 respectively. **Note:** 100 cfs = 2.832 cubic meters per second

**Figure 8.**
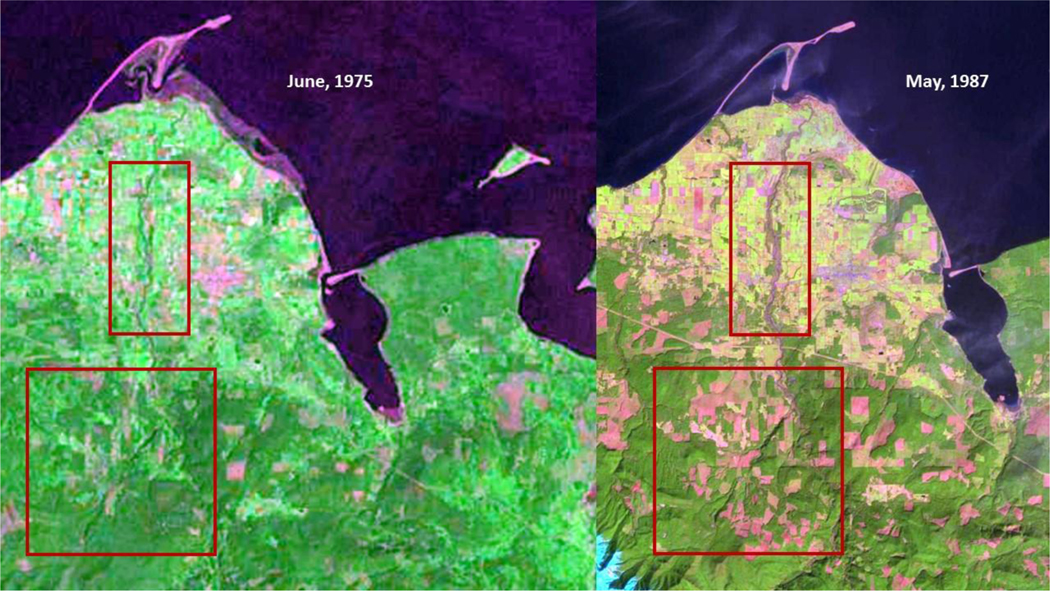
Landsat time series images: Left image - (A) June 1975, and; Right image - (B) May 1987. Left Image (A): The lower inset box outlines ‘clear-cutting’ areas occurring in the Upper Dungeness River/Wolf Creek area. The upper inset box shows increasing land use in, or close to, the river’s riparian zone. Right Image (B): The lower inset box displays further expansion of ‘clear-cutting’. The upper inset box shows the increased sediment load into the Dungeness River. Note: The ‘leaf out’ of riparian woody vegetation is just emergent in the May 1987 image

**Figure 9.**
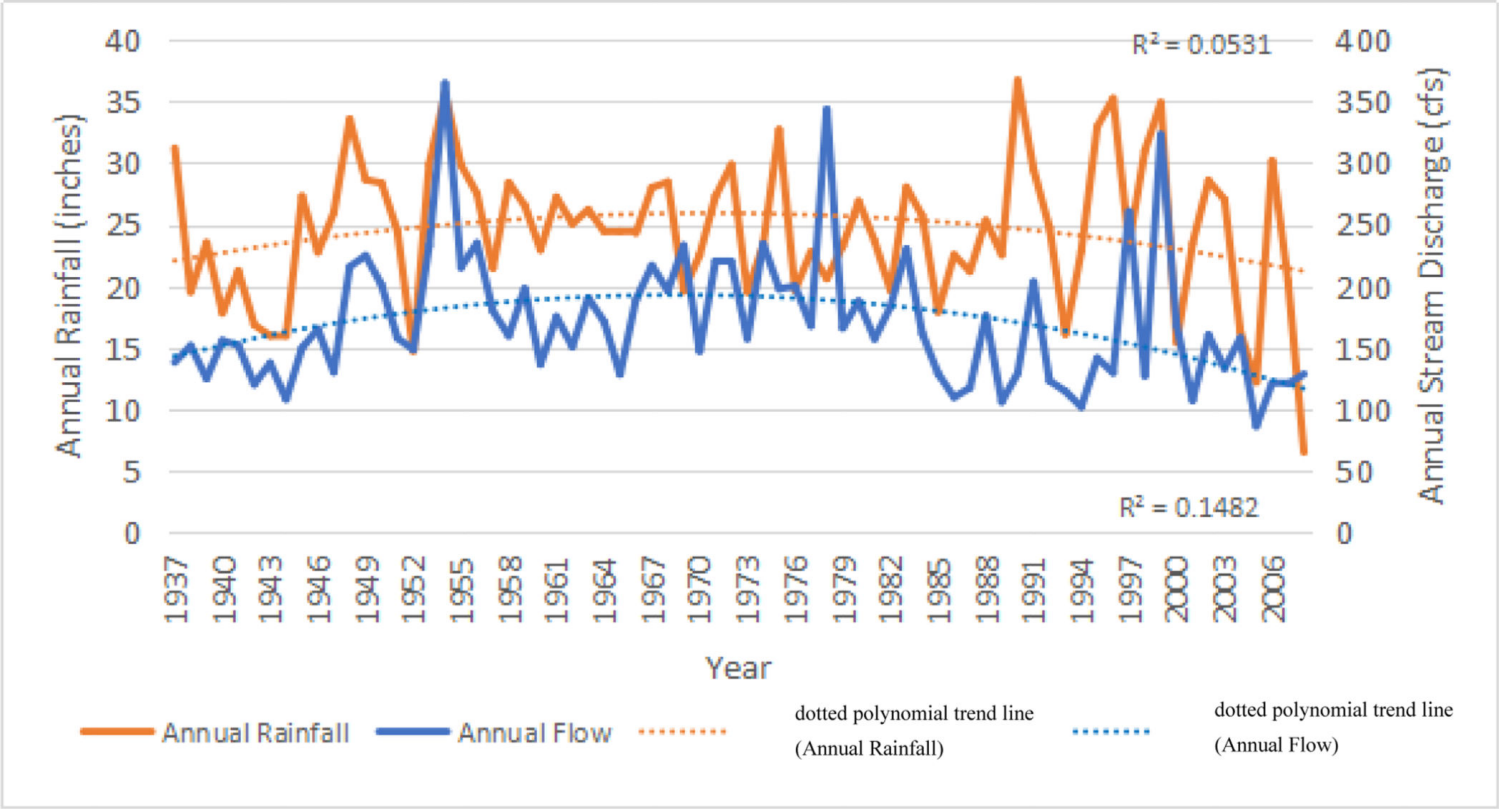
Dungeness River hydrograph of annual flow (discharge: blue line - in cubic feet per second [cfs]) and annual rainfall (red line: in inches) measured in Port Angeles WA, from 1937 to 2006. **Note:** 100 cfs = 2.832 cubic meters per second

**Figure 10.**
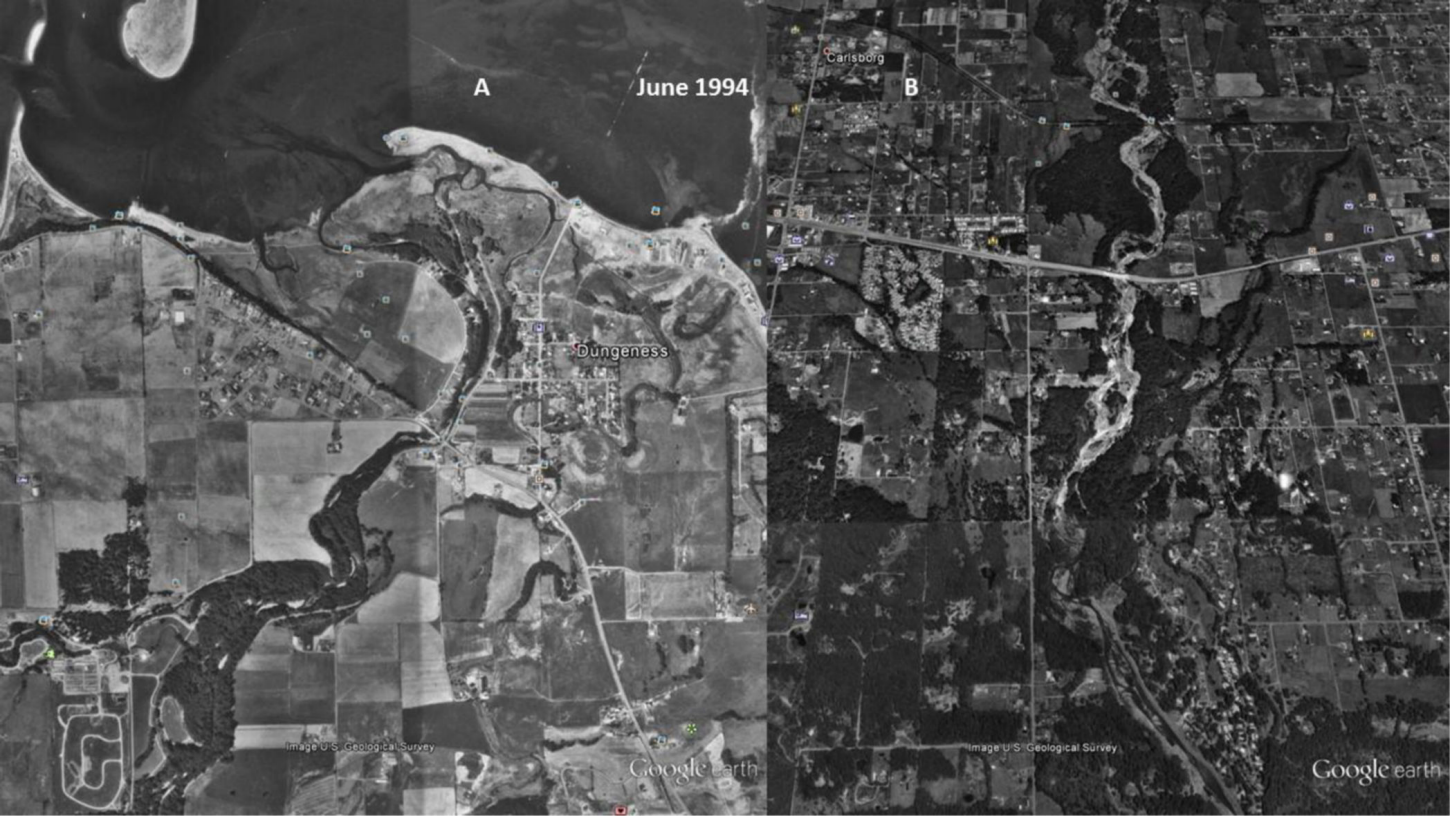
1994 USDA National Agriculture Imagery Program (NAIP) image of: Left image (A), the Lower Dungeness River, and; Right image (B), the Middle Dungeness River. Left Image (A): Sinuous and multiple outlets are at the mouth of the Dungeness River. The delta and barrier spit appear to be expanding from excess sediment. Right Image (B): Incised braided stream channel extends from the Lower to the Middle Dungeness River, north and south of Highway 101. There is very little to no re-vegetation of the point bars

**Figure 11.**
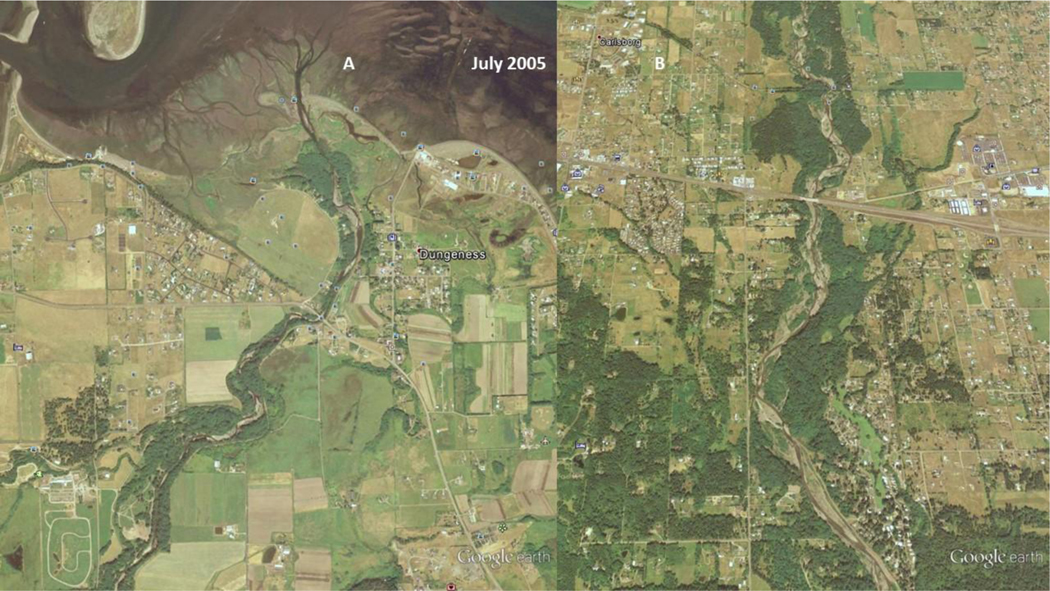
2005 USDA image of: Left image (A), the Lower Dungeness River, and; Right image (B), the Middle Dungeness River. Left Image (A): Less sinuous, almost straight, mouth of the Dungeness River, resulting from high flows cutting across the barrier beach into the Dungeness Bay. The delta appears to be expanding into the Dungeness Bay from excess sediment. Right Image (B): The stream channel along the Lower-Middle Dungeness River, north and south of Highway 101, is less braided, and the point bars are re-vegetating

The USFS land management strategy change [2] took a few years to show an impact on reducing the variability in peak flow discharge. As a result, the increased channel flow (Figures 6 and 7) of the Dungeness River breached the barrier spit in late 1994, and again in late 2001. As seen in Figure 11A, in 2005, the straightened channel created an island from the former spit [43], and transported sediment further into Dungeness Bay (**Note:** Large sand waves are visible in the upper right corner of the July 2005 image [Figure 11A].).

As seen in Figure 5 (dotted polynomial trend line), the peak stream flow (discharge) in 2005 is increasing, and the annual average discharge (and September base flow) is beginning to decrease. As seen Figure 11B, by 2005, the Dungeness River is showing some recovery of riparian functions, with increased woody riparian vegetation on the point bars. This indicates that lower stream flow rates, and less sediment, is being supplied to the lower stream channel. The slower stream flow rates assist in recharging groundwater resources.

**Figure 12.**
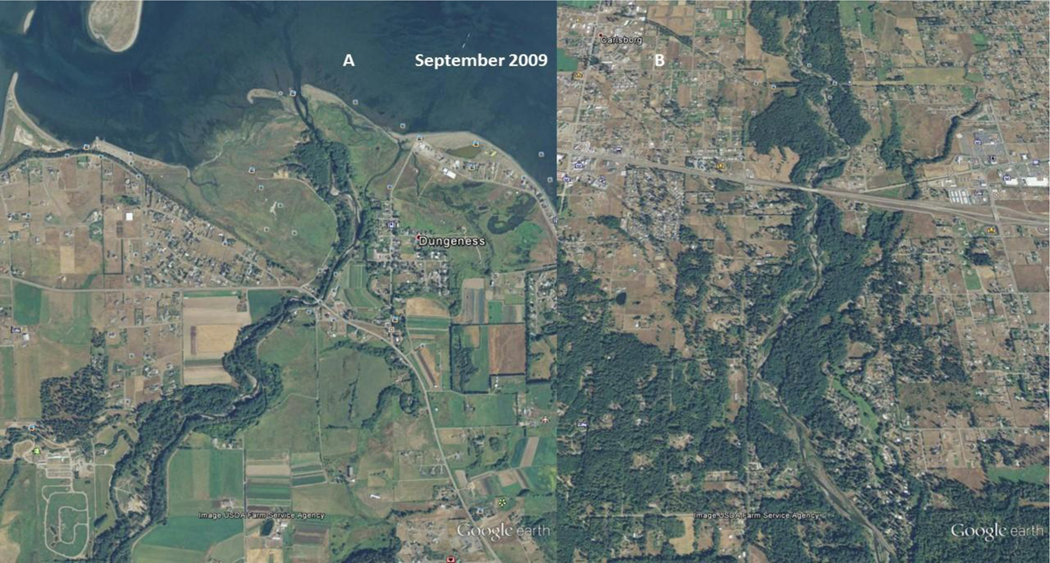
USDA 2009 National Agriculture Imagery Program (NAIP) image of: Left image (A), the Lower Dungeness River, and; Right image (B), the Middle Dungeness River. Left Image (A): The stream channel is straight, with a mid-channel bar. Riparian woody vegetation is expanding along the bend. The barrier spit is decreasing in size. Right Image (B): The Lower-Middle Dungeness River, north and south of Highway 101, has now become a single channel, and riparian vegetation appears to be stabilizing the point bars

Figure 12A shows that, in 2009, riparian vegetation at the mouth of the river is expanding laterally towards the stream channel, and out towards the shoreline. A mid-channel bar is present at the mouth of the river. The island spit appears to be decreasing in size, either because the sediment is being transported offshore, and/or sediment is being trapped further up the river channel.

Figure 12B, shows that the point bars are revegetating, and the channel is becoming more sinuous. With increased riparian functions, the vegetation will increase the side channel shear stress and facilitate trapping and deposition of both suspended and bedload material.

As seen in Figure 12A and Figure 12B, the riparian function conditions in the Dungeness River have increased to the point where the fish habitat improved. Figure 5 illustrates the higher peak flows. Figure 5 and Figure 6 display increased September base flows and average annual flows. This indicates an improvement in the physical processes (i.e., ecological functions) of the entire Dungeness River watershed.

## 4. Discussion

The DRRWG Report [39] noted that the physical processes of sediment transport must be in balance with the hydrology and sediment transport within the watershed. The USFS [2] report found the absence of stabilizing large woody debris led to decreased channel sinuosity (i.e., straightening of the channel). Past land management activities (e.g., channelization, dewatering of wetlands, etc.), loss of large wood, and floodplain access, led to increased peak flow runoff (Figure 5). Increased peak annual flow (Figure 5), would have caused the river channel to straighten, and possibly, to incise, and become disconnected from the floodplain [2]. In 1994, the USFS altered their forest management plan from ‘clear-cutting’ to commercial thinning (**Note:** There are different contributory aspects to consider in changing the forest management approach from ‘clear-cutting’ to commercial thinning, such as the magnitude of the timber harvest, the mileage of the ‘high risk’ roads in the upper portion of the watershed over time, etc.).

### TMDL

Elevated fecal coliform levels in Dungeness Bay caused the closure of shellfish beds. The state of Washington used federal and state regulations and resources to improve the fecal coliform levels, through restoration activities, and educational outreach. Once the restoration activities were implemented, the Jamestown S’Klallam Tribe, and Clallam County, monitored fecal coliform at a number of sites to determine if the restoration activities and educational outreach efforts were effective. Overall, there were lower fecal coliform concentrations (with a few mid-to late-summer spikes) and the Dungeness River showed some improvement, while the Matriotti Creek still exceeds the fecal coliform standards.

### PFC

The PFC descriptive assessment provided an overall qualitative status of the riparian-wetland area, indicating that each of the three reaches of the Dungeness River were ‘Functional-At-Risk’ (FAR) with the Upper Dungeness River Reach being in the higher end of FAR status (shown as green in Figure 1a), the Middle Dungeness River Reach being in the middle range of FAR status (shown as yellow in Figure 1a), and the Lower Dungeness River Reach being in the middle one-third of FAR status (shown as light yellow in Figure 1a). Both the Matriotti Creek and Meadowbrook Creek were in the low end of FAR status to ‘Non-Functioning’ (shown as red in Figure 1a), due to agricultural practices and urbanization.

### Remote Sensing

**Figure 13.**
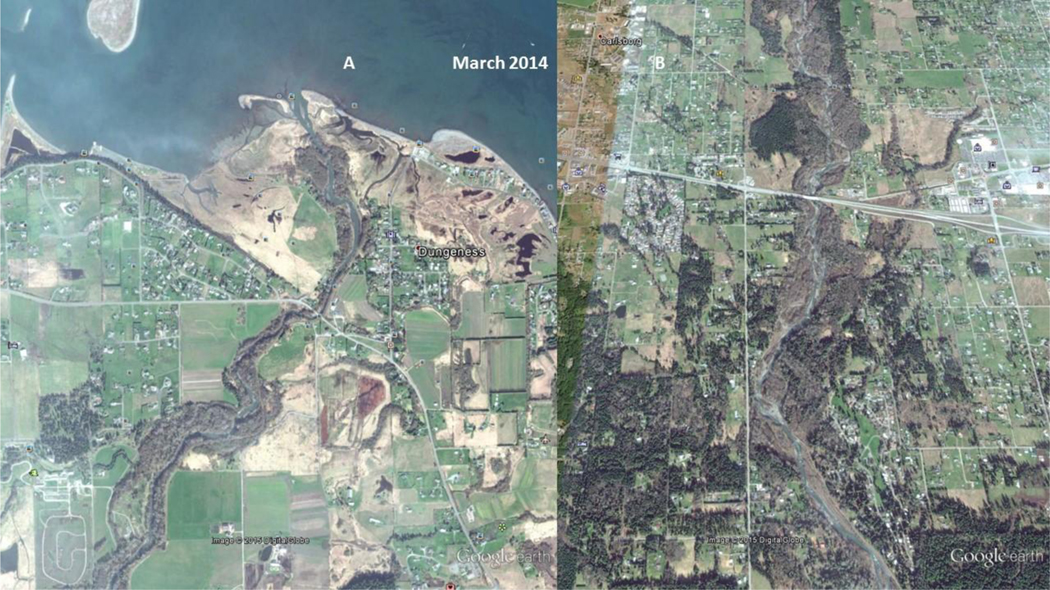
2014 USDA National Agriculture Imagery Program (NAIP) image of: Left image (A), the Lower Dungeness River, and; Right image (B), the Middle Dungeness River. Left Image (A): A single wide channel discharges into the Dungeness Bay. The barrier beach and spit have decreased in size, indicating a decrease in sediment reaching the bay. The back-beach lagoons are enlarged. Right Image (B): The riparian vegetation has stabilized the Lower-Middle Dungeness River point bars, reducing the amount of sediment reaching the Dungeness Bay

As seen in Figures 11, 12, and 13, the change in land management may have had an effect on reducing the variability in peak flow discharge. However, if riparian functions are improving, late/dry season flow should increase (Figure 5), because more water would be stored in the riparian wetlands, and also stored as groundwater (Figure 6). The downward movement of the blue (dotted) polynomial trend line in Figure 6 indicates that either management is having an effect, or there is a change in climatic conditions such as drought, and/or perhaps more precipitation as rain, with a reduced snowpack.

The evolution of the area over time was documented through remote sensing satellite photographs, which displayed the condition of the area at different times, and displayed the changes that occurred due to human activity. The USGS hydrographs displayed the water flow changes over time, and those changes were linked changes in management strategies, which occurred at discrete points in time. Also documented reports by the US federal government and the state of Washington provided a fuller context on what was happening and why these events occurred. Changes in forestry practices and conversion of forest land to agricultural use had an impact on sediment load in the area. Additional land use changes seems to have had an impact on the watershed and the riparian ecosystem. The interplay between land use, hydrology, vegetation, soil and human activity can be understood through these tools and provides the information required for a meaningful PFC assessment. Remote sensing, and PFC analysis, should track, and be consistent with monitoring and human observation.

## 5. Conclusions

Implementing a methodology to manage water quality for a riparian-wetland area requires three key elements that should be used together as a Best Management Practice (BMP): a) a visual and documentary record that illustrates the changes to an area over time, the source of the changes, and the context under which the changes occurred (remote sensing and historical reports); b) quantitative measurement and observation of the major water pollutants, sediment, nutrients, and pathogens that impact water quality, along with documentation of how corrective actions influenced water quality (TMDL and ‘before/after’ assessment of corrective actions), and; c) qualitative assessment of the riparian-wetland area that provides a description of the status of the current state of the area that can be used in planning adaptive management strategies to improve riparian-wetland functionality (PFC).

This project used remote sensing (with hydrology and historical reports), to provide a documentary record and context, TMDL targets (fecal coliform) to assess the impact of corrective actions and educational outreach on improving water quality, and PFC to describe and characterize the current status, prioritize resources and plan management actions, and provide context for quantitative measurements.

Managing water quality, by assessing functionality through PFC, enhances the sustainability of the entire ecosystem. Using a function-based approach enables everyone involved to adjust expectations as the ecosystem PFC changes in response to management options. Incorporating the pathogen (fecal coliform) TMDL implementation plan as part of a process, to achieve long-term success for stream stabilization, or restoration, and can be useful, even if it is a ‘lagging’ indicator, when used in context with other assessment (PFC), measurement (hydrology), historical (reports), and remote sensing (satellite pictures). Use of these three approaches in concert facilitates monitoring of the drivers of ecosystem function (vegetation, hydrology, soil, and landform) as a component of a watershed adaptive management plan, and is a Best Management Practices (BMPs) that can lead to increase of ecological function, and enhance the chances of improving aquatic habitat and water quality.

## Figures and Tables

**Figure 1a.**
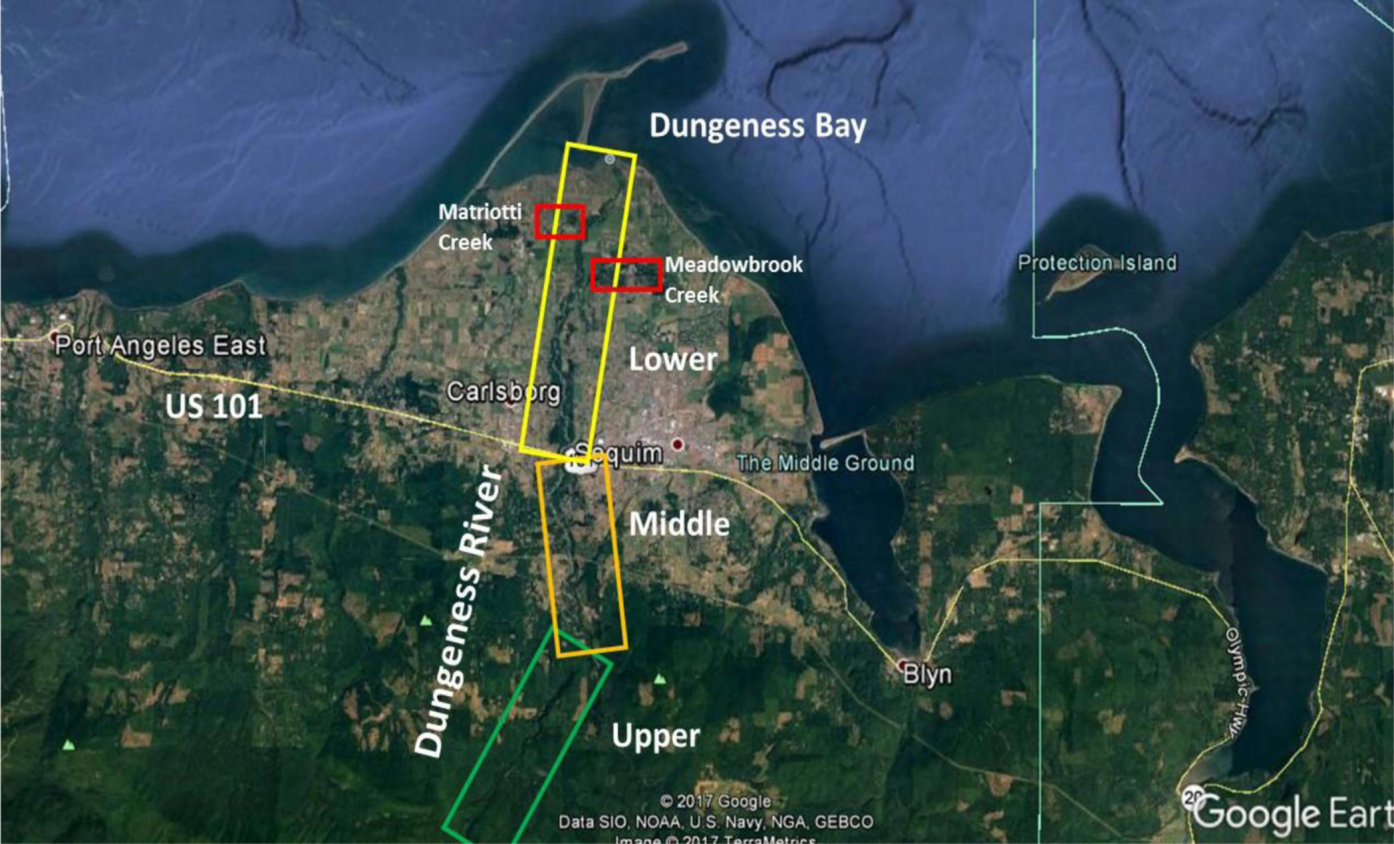
Location map of the Dungeness River study area and its reaches. The Upper Reach is located on USFS land, which was assessed at the higher end of ‘Functional-At-Risk’ (FAR), green box. The Middle Reach extends from the USFS boundary to US Highway 101 and was assessed as FAR (dark yellow box). The Lower Reach extends from US 101 to the shore zone, and was assessed in the middle one-third of FAR (light yellow box). Matriotti Creek and Meadowbrook Creek were assessed as being in the lower one-third of FAR (red boxes). Composite aerial image is from Google (Data SIO, NOAA, US Navy, GEBCO). Image @ 2017 Terra Metrics

**Figure 1b.**
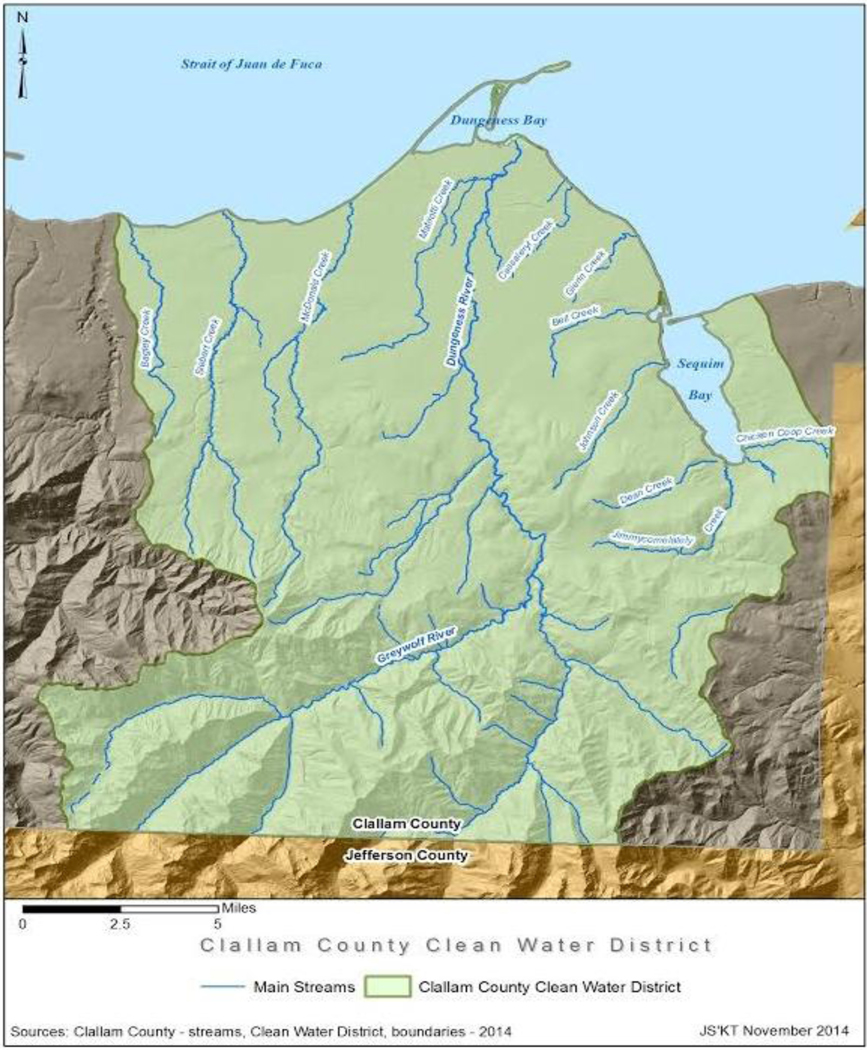
Map of the Dungeness River Watershed, including clean water district boundaries (2014). Sources: Clallam County and Jamestown S’Klallam Tribe

**Table 1.** Comparison of the Watershed Condition Framework (WCF) Protocol and the Proper Functioning Condition (PFC) Protocol

WCF	PFC	Watershed Indicators: Status
Class 1: Functioning Properly	Proper Functioning Condition	Riparian-wetland area has favorable interaction between geology, soil, water, and vegetation, etc.
Class 2: Functioning at Risk	Functional at Risk	Functional, but an existing geology, soil, water, or vegetation attribute makes the Riparian-wetland area susceptible to degradation
Class 3: Impaired Function	Non-Functioning	Riparian-wetland areas not providing adequate vegetation, landform, or large woody debris to dissipate stream energy associated with high flows; not reducing erosion, improving water quality, etc.
N/A	Unknown	Not enough data to assess

**Table 2.** Listing of Methods used in the Study, Important Characteristics, and Reason for Use

Methods Used	Characteristic/Type	Reason for Use
TMDL	Quantitative	Provides monitoring and measurement of water quality and the impact of NPS pollution (e.g., sediment, nutrients, pathogens) on aquatic communities in riparian-wetland areas.
PFC	Qualitative	Provides an assessment of stream and wetland attributes and processes (vegetation, hydrology, soil, and landform), at a point in time. Method for determining, prioritizing, and inventorying riparian resources, and providing context for quantitative data.
Remote Sensing	Visual/Graphic	Provides a visual record, over time, of change in an area, and can be used in planning to prioritize and direct resources and apply adaptive management strategies to restore the vitality of riparian-wetland areas.

## References

[1] Forest Ecosystem Management Assessment Team (FEMAT). Forest Ecosystem Management: An Ecological, Economic, and Social Assessment; US Department of Agriculture (USDA): US Forest Service (USFS), US Department Interior (DOI): Fish and Wildlife Service (FWS); National Park Service (NPS); Bureau of Land Management (BLM), US Department of Commerce (DOC): National Oceanographic and Atmospheric Administration (NOAA); National Marine and Fisheries Service (NMFS), and Environmental Protection Agency: Portland, OR, USA, July 1993., https://www.blm.gov/or/plans/nwfpnepa/FEMAT-1993/1993_%20FEMAT_Report.pdf; Accessed: 12 Oct 2019.

[2] USFS, 2012, Middle Dungeness River Subwatershed Restoration Action Plan Hood Canal Ranger District, Olympic National Forest, US Forest Service Watershed Condition Framework, FY2012 Watershed Restoration Action Plan, September 2012, 17 pgs., https://www.fs.usda.gov/Internet/FSE_DOCUMENTS/stelprdb5399955.pdf; Accessed: 12 Oct 2019.

[3] Crawford BA, March 2007, Washington State Framework for Monitoring Salmon Populations Listed under the Federal Endangered Species Act and Associated Freshwater Habitats, Produced by The Governor’s Forum on Monitoring Salmon Recovery and Watershed Health, 34 pgs., https://rco.wa.gov/documents/monitoring/Framework_Document.pdf; Accessed: 12 Oct 2019.

[4] Maringanti Chetan, Chaubey Indrajeet and Popp Jennie Popp, 2009, Development of a Multiobjective Optimization Tool for the Selection and Placement of Best Management Practices for Nonpoint Source Pollution Control, Water Resources Research, Vol. 45, 15 pgs.

[5] Smukler SM, Sanchez-Moreno S, Fonte SJ, Ferris H, Klonsky K, O’Geen AT, Scow KM, Steenwerth KL, and Jackson LE, 2010, Biodiversity and Multiple Ecosystem Functions in an Organic Farmscape; Agriculture, Ecosystems and Environment 139:80–97.

[6] Swanson S, Wyman S, and Evans C, 2015, Practical Grazing Management to Meet Riparian Objectives. J. Rangeland Applications http://journals.lib.uidaho.edu/index.php/jra/; Accessed: 12 Oct 2019.

[7] Hall RK, Guiliano D, Swanson S, Philbin MJ, Lin J, Aron JL, Schafer RJ and Heggem DT, 2014, An Ecological Function and Services Approach to Total Maximum Daily Load (TMDL) Prioritization, Journal of Environmental Monitoring and Assessment, April 2014, 186(4):2413–33, DOI 10.1007/s10661-013-3548-x, https://link.springer.com/article/10.1007%2Fs10661-013-3548-x; Accessed: 12 Oct 2019. 24435289

[8] Kozlowski D, Swanson S, Hall R, Heggem D, 2013, Linking Changes in Management and Riparian Physical Function to Water Quality and Aquatic Habitat: A Case Study of Maggie Creek, NV., EPA/600/R-13/133, USEPA Office of Research and Development, Washington, DC.

[9] Federal Water Pollution Control Act (FWPCA), 1972, Public Law 92–500, 86 Stat. 816 (Amended 1977 and 1987, Referred to as the ‘Clean Water Act’, Codified at 33 U.S.C. 1251–1387, 1988).

[10] USEPA, 2008, Guidance for Reporting Watershed Improvement under Measure SP-12 – FY 2009, December 2008,https://19january2017snapshot.epa.gov/www3/region9/water/watershed/docs/SP-12_Guidance_12-05-08.pdf; Accessed: 12 Oct 2019.

[11] USEPA, 2009, National Water Quality Inventory: Report to Congress 2004 Reporting Cycle. Office of Water, Washington, DC 20460, EPA 841-R-08–001. https://www.epa.gov/sites/production/files/2015-09/documents/2009_01_22_305b_2004report_2004_305breport.pdf; Accessed: 12 Oct 2019.

[12] Van Houtven George L., Brunnermeier Smita B., and Buckley Mark C., 2000, A Retrospective Assessment of the Costs of the Clean Water Act: 1972 to 1997. U.S. Environmental Protection Agency Office of Water Office of Policy, Economics, and Innovation, October 2000, 124 pgs.

[13] Vogan Christine R., 1996, Pollution Abatement and Control Expenditures, 1972–1994, Survey of Current Business, September, pp. 66–67.

[14] Bernhardt ES, Palmer MA, Allan JD, Alexander G, Barnas K, Brooks S, Carr J, Clayton S, Dahm C, Follstad-Shah J, Galat D, Gloss S, Goodwin P, Hart D, Hassett B, Jenkinson R, Katz S, Kondolf GM, Lake BS, Lave R, Meyer JL, O’Donnell TK, Pagano L, Powell B, Sudduth E, 2005, Synthesizing U.S. River Restoration Efforts, Science, 308:636–637. 15860611 10.1126/science.1109769

[15] Norton Douglas J., Wickham James D., Wade Timothy G., Kunert Kelly, Thomas John V., Zeph Paul, 19 May 2009, A Method for Comparative Analysis of Recovery Potential in Impaired Waters Restoration Planning, Environmental Management, 44:356–368, https://link.springer.com/article/10.1007%2Fs00267-009-9304-x; Accessed: 12 Oct 2019. 19452204 10.1007/s00267-009-9304-x

[16] Swanson Sherman, Kozlowski Donald, Hall Robert K., Heggem Daniel T., Lin John, 2017, Swanson S, Kozlowski D, Hall R, Heggem D, and Lin J. 2017. Riparian Proper Functioning Condition Assessment to Improve Watershed Management for Water quality, Journal of Soil and Water Conservation, 72-2:168–182, doi:10.2489/jswc.72.2.168., https://www.ncbi.nlm.nih.gov/pmc/articles/PMC6145829/pdf/nihms-983246.pdf; Accessed: 12 Oct 2019. 30245529 PMC6145829

[17] Giri Subhasis, Nejadhashemi A. Pouyan, and Woznicki Sean A., 2012, Evaluation of Targeting Methods for Implementation of Best Management Practices in the Saginaw River Watershed, Journal of Environmental Management, 103:24–40. 22459068 10.1016/j.jenvman.2012.02.033

[18] Veith TL, Wolfe ML, and Heatwole CD, 2004, Cost-effective BMP placement: Optimization versus targeting, Trans. ASAE, 47, 1585–1594.

[19] Messer Tiffany L., Burchell Michael R. II, Grabow Garry L., and Osmond Deanna L., 2012, Groundwater nitrate reductions within upstream and downstream sections of a riparian buffer. Ecological Engineering, 47:297–307.

[20] Wyman S, Bailey D, Borman M, Cote S, Eisner J, Elmore W, Lienard B, Leonard S, Reed R, Swanson S, Van Riper L, Westfall T, Wiley R, and Winward A, 2006, Riparian Area Management: Grazing management processes and strategies for Riparian-wetland Areas, Technical Reference 1737–20, U.S. Department of the Interior (DOI), Bureau of Land Management (BLM), National Science and Technology Center, Denver, CO., 120 pgs., https://www.blm.gov/or/programs/nrst/files/Final%20TR%201737-20.pdf; Accessed: 12 Oct 2019.

[21] Weixelman D, Zamudio D, and Zamudio K, 1996, Central Nevada riparian field guide. R4-ECOL-96–01. U.S. Department of Agriculture (USDA), U.S. Forest Service (USFS), Intermountain Region, 145 pgs.

[22] Prichard Don; Bridges Clay; Krapf Russ; Leonard Steve; and Hagenbuck Warren, 1994, Riparian Area Management: Process for Assessing Proper Functioning Condition for Lentic Riparian-Wetland Areas, Technical Reference 1737–11, Revised 1998, U.S. Department of the Interior (DOI), Bureau of Land Management (BLM)., http://www.remarkableriparian.org/pdfs/pubs/TR_1737-11.pdf; Accessed: 12 Oct 2019.

[23] Hall Robert K., and Swanson Sherman, 2009, Non-point Source Data Assessment Tools, USEPA National Non-point Source Conference, Sept. 29 - Oct. 1, Morongo Band of Mission Indians Reservation, Cabazon, CA., Abstracts with programs.

[24] Dickard M, Gonzales M, Elmore W, Leonard S, Smith D, Smith S, Staats J, Summers P, Weixelman D, and Wyman S, 2015, Riparian Area Management - Proper Functioning Condition Assessment for Lotic Areas, BLM Technical Reference 1737‐15, Second edition, 199 pgs., http://www.remarkableriparian.org/pdfs/pubs/TR_1737-15.pdf; Accessed: 12 Oct 2019.

[25] Aron JL, Hall RK, Philbin MJ, Schafer RJ, 2013, Using watershed function as the leading indicator for water quality, Water Policy, 15:850–858, DOI: 10.2166/wp.2013.111, https://iwaponline.com/wp/article/15/5/850/20078/Using-watershed-function-as-the-leading-indicator; Accessed: 12 Oct 2019.

[26] Hubert R, 2007, Transforming Sustainability: Identifying the Critical Success Factors for Sustainable Cities, Interim Report on the Sustainable Cities Research Project, 37 pgs. (Center for Sustainable Environments, Northern Arizona University, Flagstaff AZ, August 20, 2007).

[27] Swanson Sherman, Hall Robert K., Heggem Daniel T., Lin John, Kozlowski Donald and Gibson Robert J., 2012, Leading or Lagging Indicator for Water Quality Management. Abstracts with Programs, 8th National Monitoring Conference, April 30 – May 4, 2012, Portland, OR.

[28] Prichard D, Barrett H, Cagney J, Clark R, Fogg J, Gebhardt K, Hansen PL, Mitchell B, Tippy D, 1993, Riparian Area Management: Process for Assessing Proper Functioning Condition, Technical Reference 1737–9, U.S. Department of the Interior (DOI), Bureau of Land Management (BLM), Proper Functioning Condition Work Group, Revised 1995 and 1998, Bureau of Land Management Service Center, Denver, CO., 58 pgs.,https://allaboutwatersheds.org//library/general-library-holdings/Final%20TR%201737-9.pdf; Accessed: 12 Oct 2019.

[29] Prichard Don; Anderson John; Correll Cindy; Fogg Jim; Gebhardt Karl; Krapf Russ; Leonard Steve; Mitchell Brenda; and Staats Janice, 1998, Riparian Area Management, A User Guide to Assessing Proper Functioning Condition and the Supporting Science for Lotic Areas; Technical Reference TR1737–15, U.S. Department of the Interior (DOI), Bureau of Land Management (BLM), https://www.blm.gov/or/programs/nrst/files/Final%20TR%201737-15.pdf; Accessed: 12 Oct 2019.

[30] Prichard Don; Berg Forrest; Hagenbuck Warren; Krapf Russ; Leinard Robert; Leonard Steve; Manning Mary; Noble Chris; and Staats Janice, 1999, Riparian Area Management: A User Guide to Assessing Proper Functioning Condition and the Supporting Science for Lentic Areas, Technical Reference 1737–16; Revised 2003, U.S. Department of the Interior (DOI), Bureau of Land Management (BLM); https://www.blm.gov/or/programs/nrst/files/Final%20TR%201737-16%20.pdf; Accessed: 12 Oct 2019.

[31] Ward TA, Tate KW, Atwill ER, Lile DF, Lancaster DL, McDougald N, Barry S, Ingram RS, George HA, Jensen W, Frost WE, Philips R, Markegard GG, and Larson S, 2003, A comparison of three visual assessments for riparian and stream health, Journal of Soil and Water Conservation 58, 2:83–88.

[32] Burton TA, Smith SJ, and Cowley ER, 2011, Riparian Area Management: Multiple Indicator Monitoring (MIM) of Stream Channels and Streamside Vegetation, Technical Reference 1737–23, U.S. Department of the Interior (DOI), Bureau of Land Management (BLM), National Operations Center (NOC), Denver, CO., 155 pgs., https://www.fs.usda.gov/Internet/FSE_DOCUMENTS/fseprd558332.pdf; Accessed: 12 Oct 2019.

[33] USEPA, 2004. Wadeable Stream Assessment: Field Operations Manual. EPA841-B-04–004. U.S. Environmental Protection Agency, Office of Water and Office of Research and Development, Washington, DC., https://nepis.epa.gov/Exe/ZyPDF.cgi?Dockey=P100R9IQ.PDF; Accessed: 12 Oct 2019.

[34] Prichard Don; Clemmer Pam; Gorges Mark; Meyer Gretchen; Shumac Karen; Wyman Sandy; Miller Marcus, 1996, Riparian Area Management: Using Aerial Photographs to Assess Proper Functioning Condition of Riparian-Wetland Areas, Technical Reference 1737–12, Revised 1999, U.S. Department of the Interior (DOI), Bureau of Land Management (BLM), https://permanent.access.gpo.gov/lps111904/Final%20TR%201737-12.pdf; Accessed: 12 Oct 2019.

[35] USFS, 2011a, U.S. Forest Service Watershed Condition Framework, United States Department of Agriculture (USDA), United States Forest Service (USFS), FS-977, May 2011, 34 pgs., https://www.fs.fed.us/biology/resources/pubs/watershed/maps/Watershed_Condition_Framework2011FS977.pdf; Accessed: 12 Oct 2019.

[36] USFS, 2011b, U.S. Forest Service Watershed Condition Classification Technical Guide, United States Department of Agriculture (USDA), United States Forest Service (USFS), FS-978, July 2011, 49 pgs., https://www.fs.fed.us/biology/resources/pubs/watershed/maps/watershed_classification_guide2011FS978.pdf; Accessed: 12 Oct 2019.

[37] Hempleman C, and Sargeant C, 2002, Water Cleanup Plan for Bacteria in the Lower Dungeness Watershed, Total Maximum Daily Load (TMDL) Submittal Report, Washington State Department of Ecology, Southwest Regional Office, PO Box 4775, Olympia, WA 98504–7775, Publication No. 02–10-015, June 2002, 102 pgs., https://fortress.wa.gov/ecy/publications/documents/0210015.pdf; Accessed: 12 Oct 2019.

[38] Sargeant D, 2002, Dungeness River and Matriotti Creek Fecal Coliform Bacteria TMDL Study, Washington State Department of Ecology, Environmental Assessment Program Olympia, Washington 98504–7710, Publication No. 02–03-014, May 2002, https://fortress.wa.gov/ecy/publications/publications/0203014.pdf; Accessed: 12 Oct 2019.

[39] Dungeness River Restoration Work Group (DRRWG), 1997, Recommended restoration projects for the Dungeness River, Dungeness River Restoration Workgroup, 57 pgs., plus appendices.

[40] Clemmer P, 2001, Riparian area management: the use of aerial photography to manage riparian-wetland areas, BLM Technical Reference 1737–10, Denver, CO, 54 pgs.

[41] Booth D. Terrance Cox, Samuel E, and Simonds Gregg, 2007, Riparian monitoring using 2-cm GSD aerial photography, Ecological Indicators, 7: 636–648, doi:10.1016/j.ecolind.2006.07.005., http://citeseerx.ist.psu.edu/viewdoc/download?doi=10.1.1.535.2764&rep=rep1&type=pdf; Accessed: 12 Oct 2019.

[42] USFS, 1990, Land and Resource Management Plan Olympic National Forest, United States Department of Agriculture Forest Service Pacific Northwest Region. https://www.fs.usda.gov/Internet/FSE_DOCUMENTS/fsbdev3_049438.pdf; Accessed: 12 Oct 2019.

[43] Collins B, 2005, Historical geomorphology and ecology of the Dungeness River Delta and nearshore environments from the Dungeness Spit to Washington Harbor, Prepared for the Jamestown S’Klallam Tribe by the Department of Earth and Space Sciences, University of Washington, 76 pgs.

[44] Schumm SA, Harvey MD, and Watson CC, 1984, Incised Channels: Morphology, Dynamics and Control, Water Resources Publications, Littleton, CO.

[45] Rosgen D, 1996, Applied River Morphology, Wildland Hydrology, Pagosa Springs, CO., 390 pgs.

[46] Freymond Bill, Marlowe Chris, Rogers Robert W., and Volkhardt Greg, 2001, Dungeness River Chinook Salmon Rebuilding Project - Progress Report 1993–1999, Washington Department of Fish and Wildlife, January 2001, 110 pgs.

[47] Woodruff DL, Sather NK, Cullinan VI, Sargeant SL, 2009, Microbial Source Tracking in the Dungeness Watershed, Washington, Prepared for Jamestown S’Klallam Tribe in fulfillment of Task 1 (Microbial Source Tracking Study) of the Dungeness River Watershed Final Workplan for the EPA Targeted Watershed Grant Program (2004), and a Washington State Department of Ecology Centennial Grant, September 2009, 66 pgs., https://www.yumpu.com/en/document/read/54105260/microbial-source-tracking-in-the-dungeness-watershed-washington; Accessed: 12 Oct 2019.

[48] Cadmus Group, Inc., 2010, Dungeness Bay and Dungeness River Watershed Fecal Coliform Bacteria Total Maximum Daily Load: Water Quality Effectiveness Monitoring Report, Prepared for the Washington State Department of Ecology, Environmental Assessment Program, Olympia, Washington 98504–7710, Publication No. 10–03-032, May 2010, https://fortress.wa.gov/ecy/publications/documents/1003032.pdf; Accessed: 12 Oct 2019.

[49] USEPA, 2013, Revisions to the Total Coliform Rule, Federal Register, Vol. 78, No. 30, pages 10270–10365, Wednesday, February 13, 2013, https://www.govinfo.gov/content/pkg/FR-2013-02-13/pdf/2012-31205.pdf; Accessed: 12 Oct 2019.

[50] Wentz Dennis A., Brigham Mark E., Chasar Lia C., Lutz Michelle A., and Krabbenhoft David P., 2014, Mercury in the Nation’s Streams—Levels, Trends, and Implications, U.S. Geological Survey Circular 1395.

[51] Osborne JF, and Ralph SC, 1994, An aquatic resource assessment of the Dungeness River system. Phase I: Annotated bibliography and proposed study plan; Phase II: Physical channel analysis, hydrology and hydraulics; and Phase III: Fisheries habitat survey, Prepared for the Jamestown S’Klallam Tribe and the Quilcene Ranger District, Sequim, Washington.

[52] Puget Sound Cooperative River Basin Team (PSCRBT), 1991, Dungeness River Area Watershed, Report prepared by the Puget Sound Cooperative River Basin Team, Representing USDA Soil Conservation Service, USDA Forest Service, Washington State Department of Fisheries, Washington State Department of Ecology, US Environmental Protection Agency, Prepared for the Dungeness River Area Watershed Management Committee at the request of Clallam County, 132 pgs., plus appendices.

[53] Swanson S, 1996, Reading a Stream’s Need for Management, Pages 23–36, In: George MR (Tech. Coord.) Livestock Management in Grazed Watersheds - A Review of Practices that Protect Water Quality, Univ. of Calif. Davis Animal Agriculture Research Center and Univ. of Calif. Agricultural Issues Center Publication 3381, 75 pgs.

[54] Booth DB, Fox MJ, 2004, The Role of Large Woody Debris in Lowland Puget Sound Streams and Rivers, Center for Water and Watershed Studies, University of Washington (Seattle), http://www.stillwatersci.com/resources/2004boothetal_PugetLowlandLWD.pdf; Accessed: 12 Oct 2019.

[55] Czuba JA, Magirl CS, Czuba CR, Grossman EE, Curran CA, Gendaszek AS, Dinicola RS, 2011, Sediment Load from Major Rivers into Puget Sound and its Adjacent Waters, 4 pages, https://pubs.usgs.gov/fs/2011/3083/pdf/fs20113083.pdf; Accessed: 12 Oct 2019.

[56] Hynson JR, Adamus PR, Elmer JO, DeWan T, and Shields FD 1985. Environmental Features for Streamside Levee Projects. U.S. Army Corps of Engineers Waterways Experiment Station, Vicksburg, MS. Technical Report E-85–7.

[57] USFS, 2013, Planning and Layout of Small-Stream Diversions, United States Department of Agriculture (USDA), United States Forest Service (USFS), March 2013, 188 pgs., https://www.fs.fed.us/t-d/pubs/pdfpubs/pdf13251801/pdf13251801dpi100.pdf; Accessed: 12 Oct 2019.

